# KSHV hijacks the antiviral kinase IKKε to initiate lytic replication

**DOI:** 10.1371/journal.ppat.1012856

**Published:** 2025-01-17

**Authors:** Xiaoqian Wang, Zhenshan Liu, Xue Xu, Xin Wang, Zizhen Ming, Chengrong Liu, Hang Gao, Tingting Li, Qiming Liang

**Affiliations:** 1 Institute of Pediatric Infection, Immunity, and Critical Care Medicine, Shanghai Children’s Hospital, Shanghai Jiao Tong University School of Medicine, Shanghai, China; 2 Shanghai Institute of Immunology, Department of Immunology and Microbiology, Key Laboratory of Cell Differentiation and Apoptosis of Chinese Ministry of Education, Shanghai Jiao Tong University School of Medicine, Shanghai, China; 3 Joint Ph.D. Degree Program between SJTU-SM and HUJI-MED, Shanghai Jiao Tong University, Shanghai, China; 4 Department of Bone and Joint Surgery, Orthopaedic Surgery Center, The First Hospital of Jilin University, Changchun, Jilin, China; 5 State Key Laboratory of Developmental Biology of Freshwater Fish, Hunan International Joint Laboratory of Animal Intestinal Ecology and Health, Laboratory of Animal Nutrition and Human Health, Hunan Provincial Key Laboratory of Animal Intestinal Function and Regulation, College of Life Sciences, Hunan Normal University, Changsha, Hunan, China; Hannover Medical School, GERMANY

## Abstract

IKKε is a traditional antiviral kinase known for positively regulating the production of type I interferon (IFN) and the expression of IFN-stimulated genes (ISGs) during various virus infections. However, through an inhibitor screen targeting cellular kinases, we found that IKKε plays a crucial role in the lytic replication of Kaposi’s sarcoma-associated herpesvirus (KSHV). Mechanistically, during KSHV lytic replication, IKKε undergoes significant SUMOylation at both Lys321 and Lys549 by the viral SUMO E3 ligase ORF45. This SUMOylation event leads to the association of IKKε with PML, resulting in the disruption of PML nuclear bodies (PML NBs) and subsequent increase in lytic replication of KSHV. Notably, IKKε does not affect the total expression level of PML but facilitates the translocation of PML from the nucleus to the cytoplasm during KSHV lytic replication. Further experiments utilizing mutations on the SUMOylation sites of IKKε or inhibiting IKKε using BAY-985 showed that these actions no longer impact PML NBs and completely suppress the lytic replication of KSHV. These findings not only emphasize the essential role of IKKε in the life cycle of KSHV but also illustrate how KSHV exploits IKKε through SUMOylation modification to enhance its own replication process.

## Author summary

IKKε is extensively involved in host antiviral responses by regulating the production of type I IFN and the induction of some IFN-stimulated genes. However, by a cellular kinase library screening, we found that IKKε is essential for the lytic replication of KSHV. During lytic replication, IKKε undergoes robust SUMOylation on Lys231 and Lys549 by viral SUMO E3 ligase ORF45, which promotes IKKε interaction with PML, a negative regulator for KSHV lytic replication, and disrupts PML nuclear body formation by facilitating PML translocation from nucleus to cytoplasm, consequently enhancing KSHV lytic replication. Knockout of IKKε or mutations on its SUMOylation sites no longer impact PML NBs and completely suppress the lytic replication of KSHV. Our work demonstrated how KSHV utilizes ORF45 to hijacks the antiviral kinase IKKε via SUMOylation modification and benefits its own life cycle.

## Introduction

Kaposi’s sarcoma-associated herpesvirus (KSHV), also known as human herpesvirus-8, is the causative agent for Kaposi’s sarcoma (KS), the most prevalent skin cancer in individuals infected with HIV. Additionally, KSHV is implicated in various lymphoproliferative malignancies, such as primary effusion lymphoma (PEL) and multicentric Castleman’s disease [1,2]. A recent study also linked KSHV to osteosarcoma in the Xinjiang population [3]. Similar to other herpesviruses, the lifecycle of KSHV involves both latency and lytic replication. While latent infection is highly associated with KS tumors, spontaneous lytic replication frequently occurs in KS lesions, leading to the production of inflammatory cytokine and potentially aiding in KSHV-induced tumorigenesis [1]. KSHV encodes over 80 open reading frames (ORFs) and 12 viral pre-microRNAs that play key roles in various cellular processes, creating an optimized microenvironment for viral replication and tumorigenesis [4].

Among the KSHV-encoded ORFs, ORF45 plays a significant role in the KSHV life cycle [5]. ORF45 is an immediate-early lytic gene and a tegument structural gene critical for various functions [5,6]. It has been found to inhibit the phosphorylation of IRF7, thereby suppressing type I interferon (IFN) production [7,8]. Additionally, the cellular receptor human NLRP1 can recognize KSHV ORF45 during infection, leading to the initiation of inflammasome assembly and activation [9]. ORF45 interacts with ORF33, another tegument protein, to enhance ORF33 stability by reducing its ubiquitination through Ubiquitin-specific-processing protease 7 (USP7, or HAUSP) [10]. It also interacts with the kinesin-2 motor protein KIF3A to aid in the transportation of the capsid-tegument complex along microtubules [11]. The mono-ubiquitination of ORF45 is essential for viral assembly and egress by anchoring KSHV particles to lipid rafts [12]. Moreover, ORF45 activates cellular Extracellular signal-regulated kinase (ERK) and Ribosomal Protein S6 Kinase (RSK) signaling by forming a complex with ERK and RSK, thus preventing their dephosphorylation [13–15]. ORF45 also acts as a SUMO-interaction motif (SIM)-dependent SUMO E3 ligase to promote RSK1 SUMOylation, necessary for RSK1-eIF4B association and activation of the translation initiation complex [16,17]. The activation of the ERK-RSK pathway by ORF45 further phosphorylates c-Fos and eIF4B, leading to enhanced transcription or translation efficiency of viral proteins during lytic replication [18,19]. Deletion of ORF45 or disruption of its SUMO E3 ligase activity significantly hinders KSHV lytic replication [17,20].

The maintenance of KSHV latency and efficient lytic replication crucially rely on the close interaction with the host SUMO modification system. SUMO modification is indispensable for KSHV latency maintenance and cell transformation [21,22]. LANA interacts with SUMO2 through two SIMs to recruit the chromatin remodeling proteins and transcriptional factors like TRIM28 and HIF-1α [23]. LANA itself is SUMOylated at Lys1140, essential for episome maintenance and RTA inactivation [23]. vIRF3 disrupts the SUMOylation of pRb, p107, and p130, leading to the inhibition of Rb’s function and subsequently enhanced cell growth [24]. It also contains a SIM and promotes the SUMOylation and ubiquitination of PML, resulting in PML degradation and contributing to KSHV-mediated cell transformation [25]. SUMO modification is also vital for efficient KSHV lytic replication. RTA acts as a SIM-dependent SUMO-targeting ubiquitin E3 ligase (STUbL), and its STUbL activity is crucial for RTA transactivation during KSHV lytic cycle [26]. K8 functions as a SIM-dependent SUMO E3 ligase targeting both IRF3 and IRF7 to reduce type I IFN production [27]. SUMOylation of K8 at Lys158 is essential for the transcriptional suppressive activity [28]. Our previously studies have shown that KSHV ORF45 is a SIM-dependent SUMO E3 ligase, and mutations in its SIMs completely block KSHV lytic replication [17]. ORF45 catalyzes the SUMOylation of RSK1 at Lys110, Lys335, and Lys421, facilitating the interaction between RSK1 and its downstream substrate eIF4B, promoting the phosphorylation of eIF4B and efficient translation of viral mRNA [16]. However, it remains unclear whether ORF45-mediated SUMOylation regulates other viral or cellular proteins in the KSHV life cycle.

IκB kinase ε (IKKε), also known as IKKi, is a non-canonical IκB kinase that plays a significant role in host antiviral responses by regulating the production of interferons (IFNs) and the induction of certain IFN-stimulated genes (ISGs) [29]. It has been observed that IKKε and TBK1 share a similar substrate phosphorylation motif in vitro [30]; however, genetic knockout studies have shown that TBK1, not IKKε, is crucial for IFN induction in response to viral infections [31,32]. While TBK1 is constitutively expressed in most cell types, the expression of IKKε is induced by type I IFNs and various inflammatory cytokines, and its expression is restricted to specific cell types [33,34]. Upon type I IFN treatment, IKKε, but not TBK1, can phosphorylate STAT1 on Ser708, leading to the expression of a subset of ISGs [32,35]. Furthermore, in response to DNA damage, IKKε undergoes robust SUMOylation at Lys231 by the cellular SUMO E3 ligase TOPORS, resulting in IKKε nuclear translocation, particularly within Promyelocytic leukemia (PML) nuclear bodies (PML NBs), and activation of the anti-apoptotic function of NF-κB [36,37]. KSHV miR-K12-11 has been reported to reduce IKKε expression to dampen IFN signaling, aiding in maintaining KSHV latency [38]. IKKε and IKKβ work together to coordinate NF-κB activation to support KSHV latent infection [39]. Additionally, IKKε plays a crucial role in vGPCR-mediated tumorigenesis [40]. However, the specific role of IKKε in the KSHV lytic cycle is not yet fully understood.

PML NBs, also called nuclear domain 10 (ND10), are large protein complexes composed of a PML protein core and other components like SP100 and DAXX. These structures play crucial roles in various cellular processes, including chromatin remodeling, transcriptional regulation, DNA damage responses, tumor suppression, apoptosis, and antiviral responses [41]. PML, also known as TRIM19, is the key organizing molecule within PML NBs. It contains one SIM and undergoes substantial SUMOylation at specific lysine residues (Lys65, Lys160, and Lys490), which are essential for PML-NB assembly through interactions with SUMO-modified proteins [42]. Previous studies have revealed that viral proteins like vIRF3 (also known as LANA2) and ORF75 from KSHV have the ability to counteract PML-NBs, aiding in efficient lytic replication [43,44]. Non-PML NB resident SP100 functions as a negative regulator of polycomb repressive complex-2 (PRC2) recruitment and KSHV actively escape PML NB silencing mechanisms to promote establishment of latent chromatin [45]. In this study, a small molecule inhibitor screen targeting host kinases identified IKKε as a key player for KSHV lytic replication. IKKε is significantly SUMOylated by the viral SUMO E3 ligase ORF45 at specific lysine residues (Lys231 and Lys549). Mutations on these lysine residues completely inhibit KSHV lytic replication. The ORF45-mediated SUMOylation of IKKε promotes the association between IKKε and PML, resulting in the disruption of PML NBs and facilitating efficient KSHV lytic replication.

## Results

### IKKε is required for KSHV lytic replication

**Fig 1:**
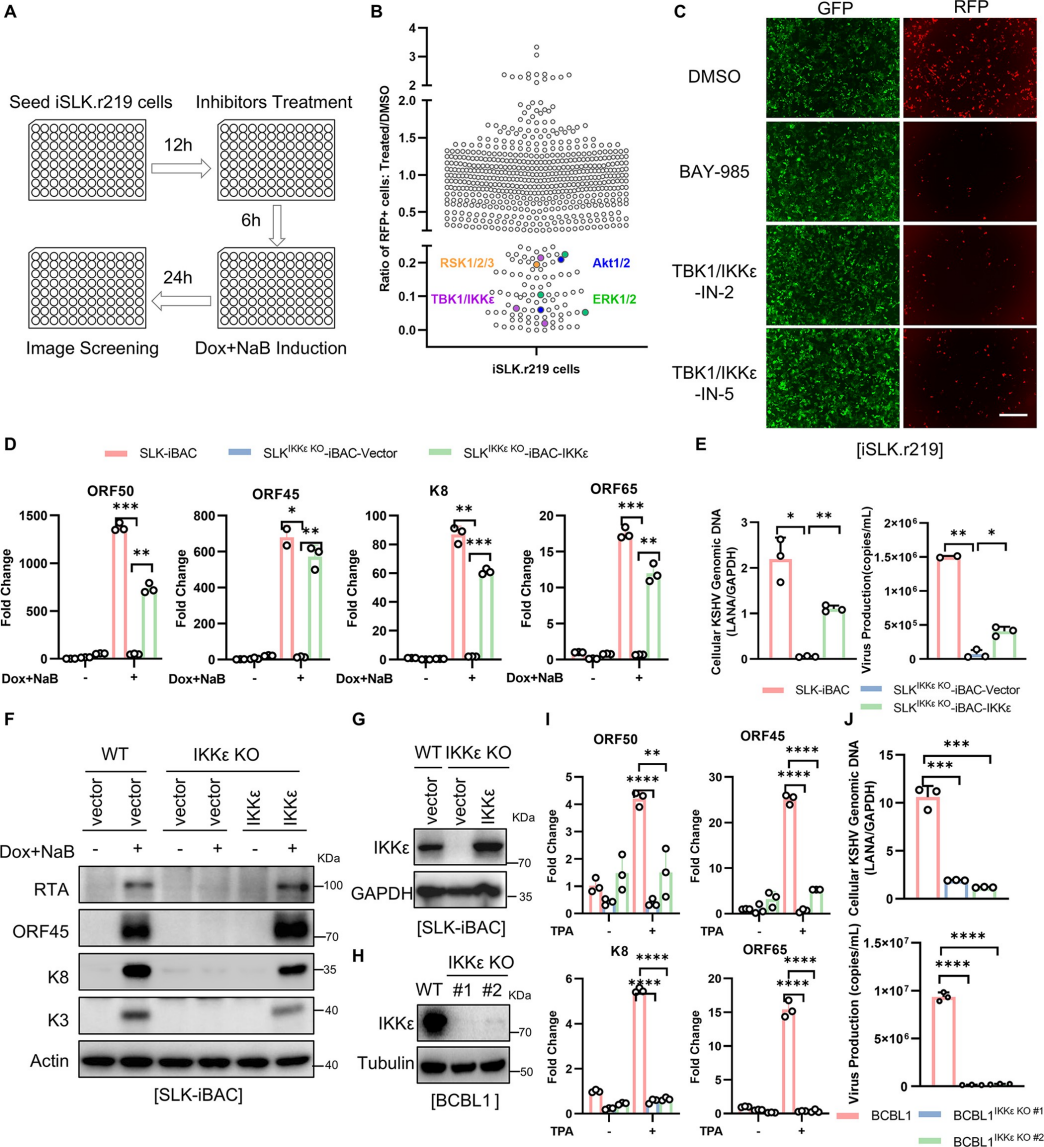
IKKε is required for KSHV lytic replication. (*A*) Screen workflow of the human kinase library for KSHV lytic replication. The iSLK.r219 cells were pre-treated with 10 μM kinase inhibitors for 6 hours, and induced by 2 μg/mL doxycycline (Dox) and 1 mM sodium butyrate (NaB) for 24 hours. The GFP and RFP images were captured by Keyence Automated High-Resolution microscope (BZ-X800). (*B*) Summary of the screened results. Each dot represents a kinase inhibitor, its effect on KSHV lytic replication was quantified with the ratio of RFP to GFP fluorescence intensities by automated image cytometer analysis from Keyence microscope (BZ-X800) and showed by normalizing the ratio to control DMSO-treated sample. (*C*) Inhibitors targeting TBK1 and IKKε limited KSHV lytic replication. The iSLK.r219 cells were treated with indicated inhibitors (10 μM) for 6 h, followed by treatment with Dox (2 μg/mL) and NaB (1 mM) for 24 h. The GFP and RFP images were captured by Keyence microscope (BZ-X800). Scale bar, 100 μm. (*D*-*G*) IKKε is required for KSHV lytic replication in SLK-iBAC cells. SLK-iBAC cells and SLK^IKKε KO^-iBAC cells stably complemented with vector or IKKε were treated with Dox/NaB for 48 h to induce KSHV lytic replication. Indicated viral gene expressions at RNA level were determined by qRT-PCR (*D*). Total DNA was isolated from the cell lysates or the culture supernatant of indicated cells and viral genomic DNA was quantified by qPCR (*E*). Cell lysates were collected and the viral protein expression (*F*) or IKKε levels (*G*) were determined by immunoblotting (IB) with indicated antibodies. (*H-J*) IKKε is required for KSHV lytic replication in BCBL1 cells. BCBL1 and BCBL1^IKKε KO^ (2 clones) were treated with TPA for 72 h to induce KSHV lytic replication. Cell lysates were collected and subjected to IB with indicated antibodies (*H*). Indicated viral gene expressions at RNA level were determined by qRT-PCR (*I*). Total DNA was isolated from the cell lysates or the culture supernatant of indicated cells and viral genomic DNA was quantified by qPCR (*J*). Data represent the means of three independent experiments; Mean ± SD; *p < 0.05, **p < 0.01, ***p < 0.001, and ****p < 0.0001 by one-way ANOVA in (*D-E*, *I-J*).

As an intracellular parasite, KSHV has to modulate cellular kinases to evade immune surveillance and maintain efficient replication. To determine which cellular kinases are critical for KSHV reactivation, we performed an inhibitor screen targeting cellular kinases. Over 650 inhibitors targeting 248 cellular kinases were included in this screen. For the screen, we utilized KSHV-infected iSLK (iSLK.r219) cells that carry latent KSHV and constitutively express GFP, whereas lytic reactivation drives the RFP expression since it is under the control of a lytic promoter [46]. To minimize false discoveries, multiple compounds targeting the same cellular kinase were included with each compound tested in triplicate along with robust statistical analysis in the primary screen. The iSLK.r219 cells were pre-treated with 10 μM kinase inhibitor or DMSO control for 6 hours and induced by doxycycline (Dox) and sodium butyrate (NaB) to trigger lytic reactivation. At 24 hours post-reactivation, the GFP and RFP images were captured and the level of lytic replication was quantified by comparing the fluorescence intensities of RFP to GFP by Keyence Automated High-Resolution microscopy (**Fig 1A**). The results indicated that over 80 inhibitors targeting 46 kinases significantly inhibited KSHV lytic replication by more than 80%, while 14 inhibitors targeting 12 kinases enhanced KSHV lytic replication by more than 2-fold (**Fig 1B**). These findings were further validated by measuring viral lytic gene expression through quantitative RT-PCR. Specific inhibitors, such as SR-4835 targeting CDK and MK8722 targeting AMPK, were identified to abolish KSHV lytic replication, whereas inhibitors like E-Necrosulfonamide targeting MLKL and AUDA targeting soluble epoxide hydrolase (sEH) promoted KSHV lytic replication in SLK-iBAC cells, confirming the inhibitor screening results (**S1A and S1B Fig**). Consistent with the previous reports, the inhibition of Akt with Oridonin, inhibition of ERK with Notoginsenoside R1 [47,48], and inhibition of RSK with LJI308 [14,18] were observed to modulate KSHV lytic replication in iSLK.r219 cells (**S1A Fig**). These results shed light on the critical role of specific cellular kinases in the reactivation of KSHV.

Among these kinase inhibitors, three top kinase inhibitors—BAY-985, TBK1/IKKε-IN-2, and TBK1/IKKε-IN-5—targeting the type I IFN kinases TBK1 and IKKε were found to significantly reduce KSHV lytic replication (**Fig 1C**). To further investigate the necessity of these kinases in the KSHV lytic cycle, individual knockout models of TBK1 and IKKε were generated in SLK-iBAC cells by CRISPR-Cas9. Interestingly, the knockout of TBK1 showed minimal effects on KSHV lytic replication and progeny virus production (**S1C and S1D Fig**). Conversely, the knockout of IKKε resulted in a complete abolishment of the lytic gene expression, viral DNA replication, and progeny virus production of KSHV (**Fig 1D to 1G**), suggesting these inhibitors reduce KSHV lytic replication by inhibiting IKKε rather than TBK1. Furthermore, a complementation assay indicated that stably expression of IKKε, but not control vector, rescued KSHV lytic replication in SLK^IKKε KO^-iBAC cells (**Fig 1D to 1G**), confirming the role of IKKε for KSHV lytic replication. Additionally, the knockout of IKKε also led to a reduction in KSHV lytic replication in BCBL1 cells following TPA treatment (**Fig 1H to 1J**). These results demonstrated that the innate immune kinase IKKε is required for KSHV lytic replication.

### IKKε is SUMOylated by ORF45 during KSHV lytic replication

**Fig 2:**
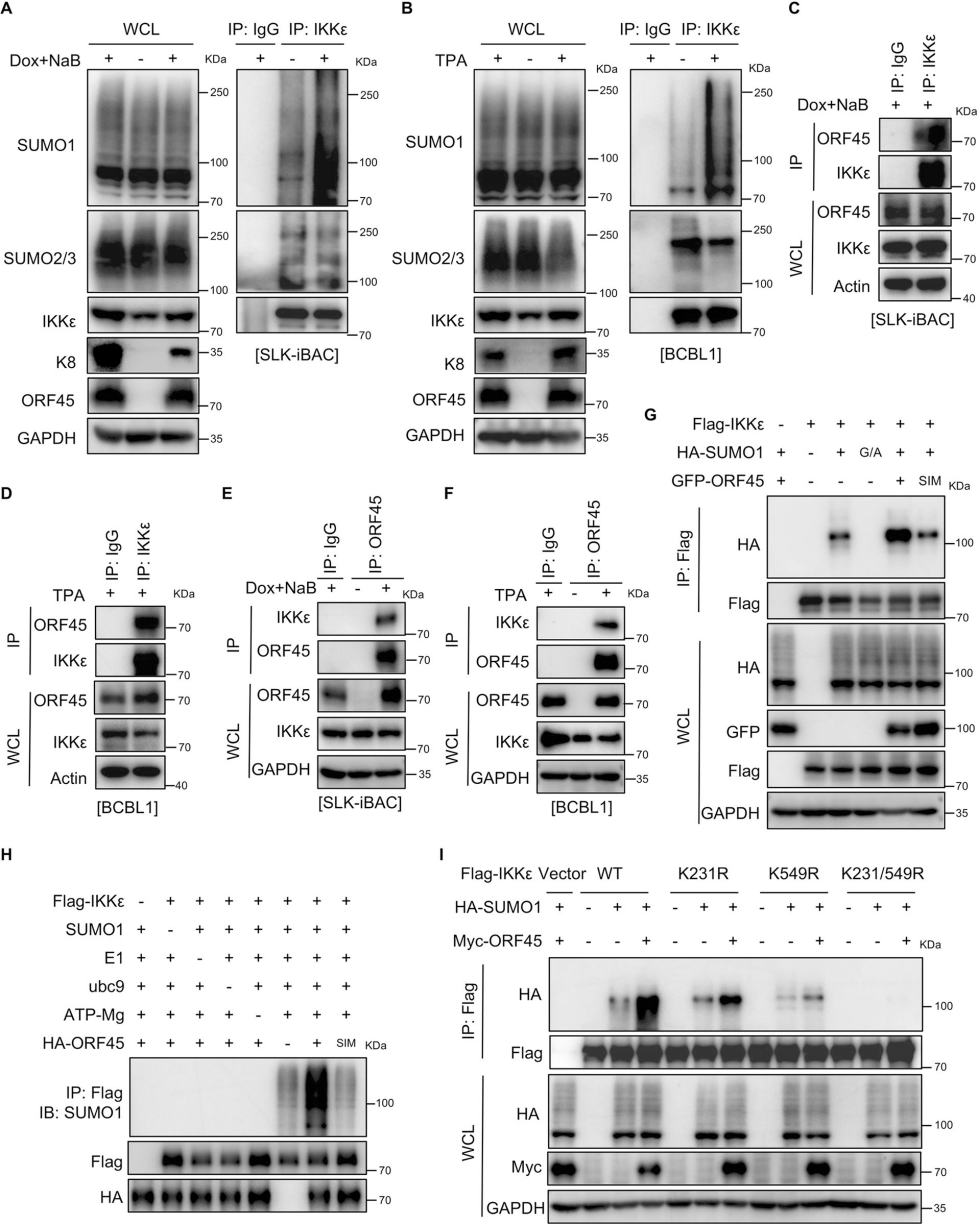
IKKε is SUMOylated by ORF45 at Lys231 and Lys549 during KSHV lytic replication. (*A*-*B*) KSHV lytic replication promotes IKKε SUMOylation. SLK-iBAC cells (*A*) and BCBL1 cells (*B*) were induced with Dox/NaB or TPA, respectively, and cell lysates were collected at 48–72 h post-induction, followed by immunoprecipitation (IP) under denature condition and IB with indicated antibodies. (*C*-*F*) ORF45 interacts with endogenous IKKε during KSHV lytic replication. SLK-iBAC cells (*C* and *E*) and BCBL1 cells (*D* and *F*) were induced with Dox/NaB or TPA, respectively, and cell lysates were collected at 48–72 h post-induction, followed by IP and IB with indicated antibodies. (*G*) ORF45 promotes IKKε SUMOylation in a SIM-dependent manner. HEK293T cells were co-transfected with indicated plasmids and cell lysates were subjected to IP under denature condition and IB with indicated antibodies. (*H*) ORF45 catalyzes IKKε SUMOylation *in vitro*. Purified Flag-IKKε was incubated with SAE1/UBA2 (E1), Ubc9 (E2), SUMO1, ATP/Mg and purified HA-ORF45 or HA-ORF45^SIM^ in SUMO reaction buffer. The SUMOylation of IKKε was detected with indicated antibodies. (*I*) Lys231 and Lys549 are the primary SUMOylation sites of IKKε by ORF45. HEK293T cells were co-transfected with indicated plasmids and cell lysates were subjected to IP under denature condition and IB with indicated antibodies.

IKKε is a serine/threonine kinase known for its involvement in the production of type I IFN as well as the induction of ISGs [29]. It is phosphorylated during activation and undergoes SUMOylation by the cellular E3 ligase TOPORS in response to DNA damage [29,37]. In the context of KSHV lytic replication, we observed robust SUMOylation of IKKε in both SLK-iBAC cells and BCBL1 cells (**Fig 2A and 2B**). Given the critical role of cellular SUMOylation in KSHV lytic replication and the presence of multiple KSHV-encoded genes that facilitate the SUMOylation process [21], we investigated how KSHV regulates IKKε SUMOylation during the lytic cycle. Through co-expression experiments in HEK293T cells involving IKKε, SUMO1, and various KSHV-encoded SUMO E3 ligases including K8 [27], RTA [26], LANA [23], and ORF45 [17], we discovered that ORF45 significantly enhanced IKKε SUMOylation, while K8, LANA, or RTA had minimal or no effect (**S2A Fig**). Our previous study has shown that overexpressed ORF45 interacts with IKKε in HEK293T cells [8], and this interaction was also detected at the endogenous level in both SLK-iBAC cells and BCBL1 cells during KSHV lytic replication (**Fig 2C to 2F**). Further molecular mapping pinpointed the region of IKKε (amino acid 300–500 and amino acid 500–716) responsible for the interaction with ORF45 (**S2B Fig**). Unlike the wild-type ORF45, mutations in both SIM1 and SIM2 (ORF45^SIM^) abrogated its ability to promote IKKε SUMOylation (**Fig 2G**). The SUMO1 G/A mutant cannot be ligated to lysine residues and served as a negative control for SUMOylation modification of IKKε (**Fig 2G**). *In vitro* experiments also confirmed that recombinant wild-type ORF45, but not its SIM mutant, catalyzed IKKε SUMOylation (**Fig 2H**), suggesting ORF45 as the SIM-dependent SUMO E3 ligase for IKKε. Moreover, unlike in iSLK-BAC16 cells, the level of IKKε SUMOylation did not change in iSLK-BAC16-ORF45^stop^ cells (**S2C Fig**), demonstrating that ORF45 is not only necessary but also sufficient for IKKε SUMOylation during KSHV lytic replication. These results indicated that IKKε is a substrate for SUMO E3 ligase ORF45.

Next, we determined the specific amino acids of IKKε that are SUMOylated by KSHV ORF45. It was initially found that IKKε mainly undergoes SUMOylation at Lys231 (K231) by the cellular E3 ligase TOPORS in response to DNA damage [37]. Interestingly, mutating Lys231 to arginine (K231R) did not abolish ORF45-mediated SUMOylation of IKKε (**Figs 2I** and **S2D**), indicating the involvement of other lysine residues in this process. To identify these additional sites, a series of mutations were introduced to different lysine residues of IKKε, and their effects on IKKε SUMOylation by ORF45 were assessed. The results revealed that double mutations on both Lys231 and Lys549 to arginine (K231R/K549R) completely prevented IKKε SUMOylation by ORF45 (**Fig 2I**). In contrast, mutations on other lysine residues had minimal to no effect (**Figs 2I** and **S2D**). Notably, individual mutation at either K231R or K549R mutation did not abolish IKKε SUMOylation by ORF45 (**Fig 2I**), underscoring the significance of both amino acids as primary SUMOylation sites for IKKε by ORF45. These findings conclusively demonstrate that the viral SUMO E3 ligase ORF45 catalyzes IKKε SUMOylation on both Lys231 and Lys549 during KSHV lytic replication.

### IKKε SUMOylation is required for KSHV lytic replication and progeny virus production

**Fig 3:**
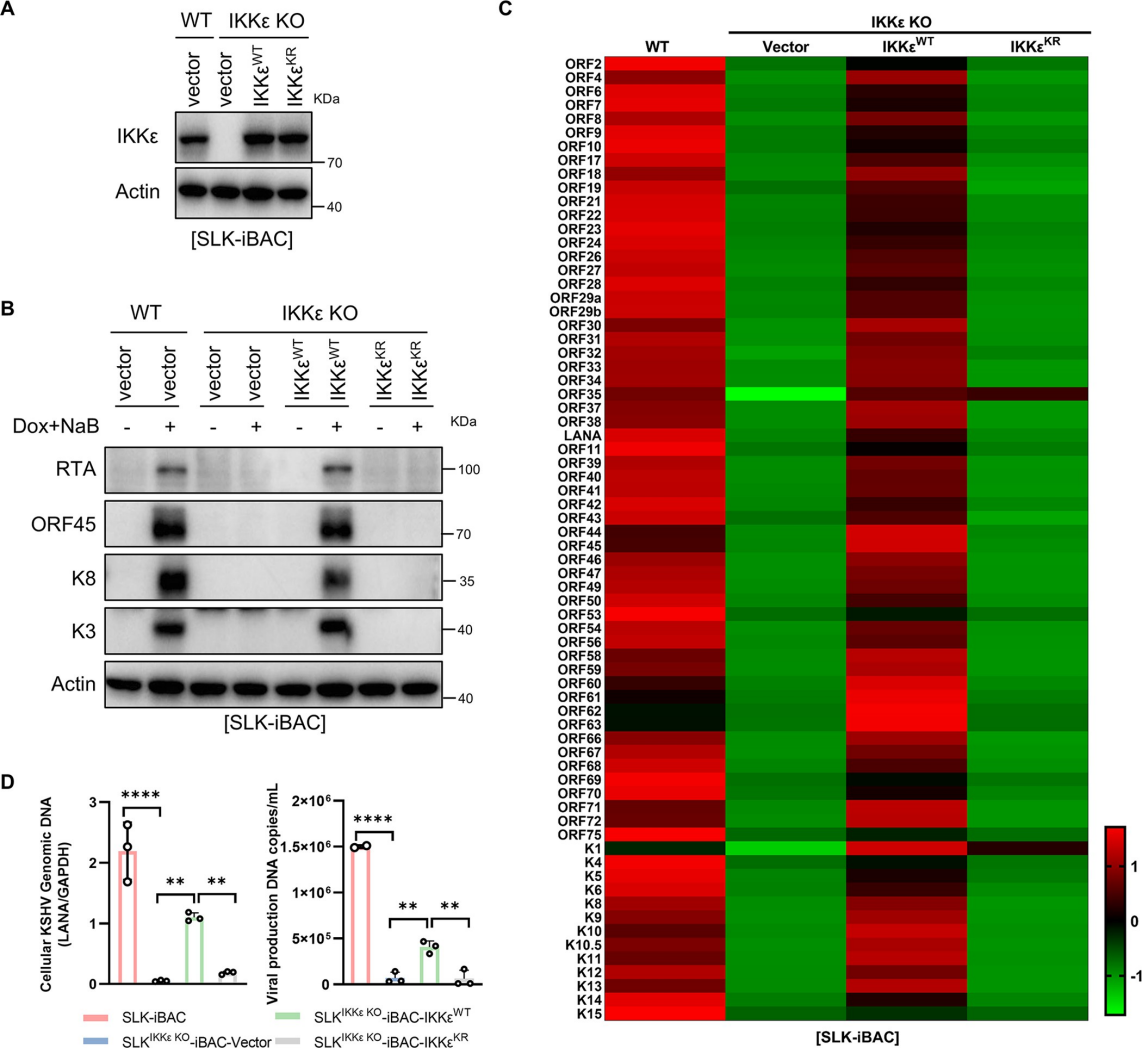
IKKε SUMOylation is required for KSHV lytic replication in SLK-iBAC cells. (*A*) Cell lysates from SLK-iBAC or SLK^IKKε KO^-iBAC stably complemented with IKKε^WT^, IKKε^KR^, or control vector were subjected to IB with indicated antibodies. (*B-C*) IKKε SUMOylation is required for viral gene expressions at both protein and RNA levels during KSHV lytic replication. SLK-iBAC and SLK^IKKε KO^-iBAC cells stably complemented with IKKε^WT^, IKKε^KR^, or control vector were treated with Dox and NaB to induce KSHV lytic replication. Cell lysates were collected at indicated time points and subjected to IB with indicated antibodies (*B*). Total RNA was extracted, reverse-transcribed into cDNA, and used for KSHV whole-genome qPCR array analysis at 48 h post-induction. The ΔCT values for each primer set were calculated and converted to a heatmap using R (*C*). (*D*) IKKε SUMOylation affects KSHV viral genomic DNA replication and progeny virus production. Viral genomic total DNA was isolated from indicated cell lines with Dox/NaB treatment for 48 h and quantified by qPCR. Progeny virus was isolated from the culture supernatant of indicated cell lines with Dox/NaB treatment for 72 h and viral genomic DNA was quantified by qPCR. Data represent the means of three independent experiments; Mean ± SD; **p < 0.01 and ****p < 0.0001 by one-way ANOVA in (*D*).

**Fig 4:**
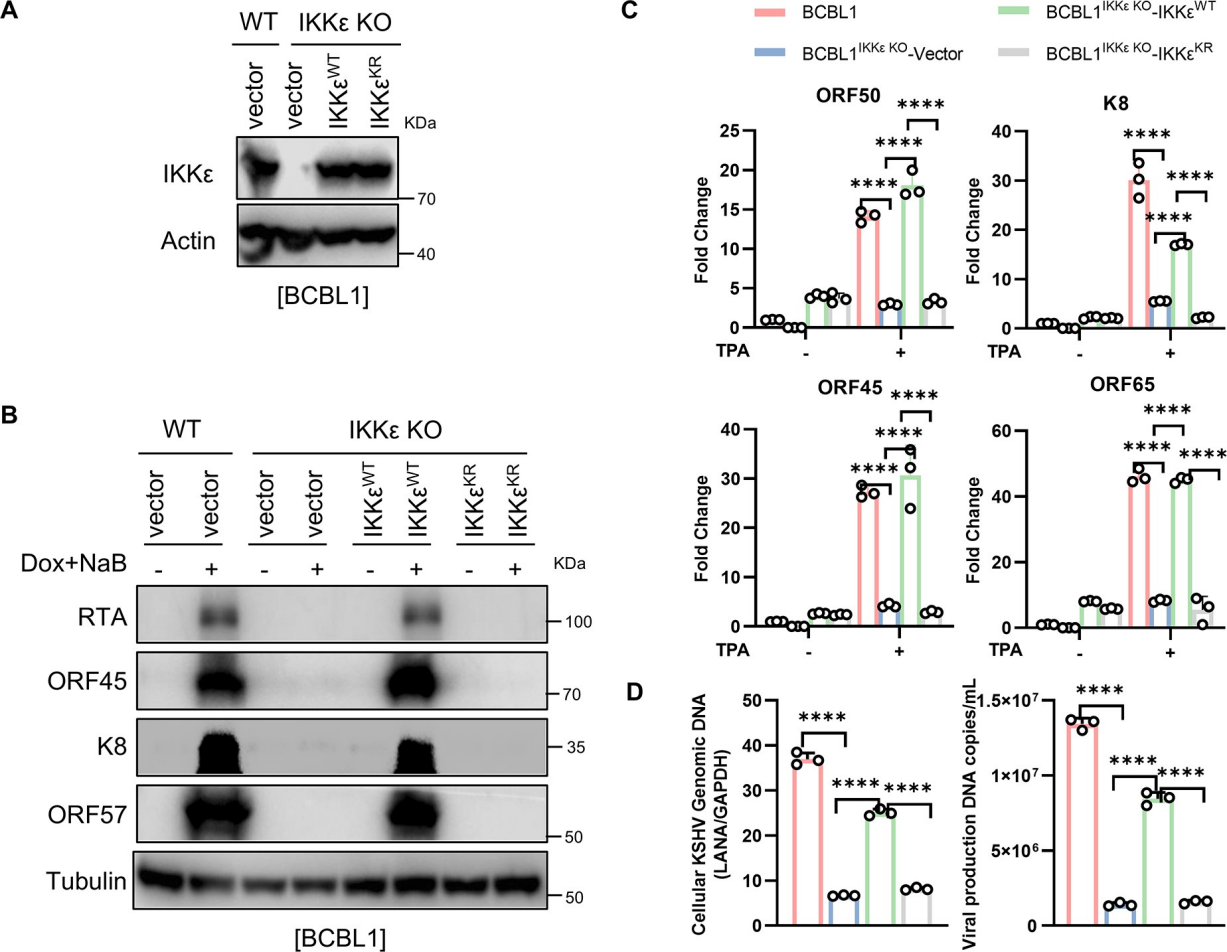
IKKε SUMOylation is required for KSHV lytic replication in BCBL1 cells. (*A*) Cell lysates from BCBL1 or BCBL1^IKKε KO^ stably complemented with IKKε^WT^, IKKε^KR^, or control vector were subjected to IB with indicated antibodies. (*B*-*D*) IKKε SUMOylation is required for KSHV lytic replication in BCBL1 cells. Indicated BCBL1 were treated with TPA for 72 h and cell lysates were collected and subjected to IB with indicated antibodies (*B*). Indicated viral gene expressions at RNA level were determined by qRT-PCR (*C*). Total DNA was isolated from the cell lysates or the culture supernatant of indicated cells and viral genomic DNA was quantified by qPCR (*D*). Data represent the means of three independent experiments. Mean ± SD; **p < 0.01 and ****p < 0.0001 by one-way ANOVA in (*C-D*).

Since IKKε is highly SUMOylated by ORF45 during KSHV lytic replication, we next investigated whether this post-translational modification of IKKε is required for KSHV lytic replication. We complemented SLK^IKKε KO^-iBAC cells with wild-type IKKε (IKKε^WT^), IKKε^K231R/K549R^ (IKKε^KR^), or vector control by lentiviruses and assessed the efficiency of KSHV lytic replication following treatment with Dox and NaB (**Fig 3A**). Immunoblot analysis revealed that in SLK^IKKε KO^-iBAC cells, the expression levels of immediate-early genes (RTA, K8, and ORF45) and an early gene (K3) were significantly restored by IKKε^WT^ at 48 hours post-induction, resembling levels observed in wild-type SLK-iBAC cells (**Fig 3B**). However, the SUMOylation-deficient mutant IKKε^KR^ failed to support the expression of these viral proteins, highlighting the critical role of IKKε SUMOylation in KSHV lytic replication (**Fig 3B**). Furthermore, a comparison of KSHV viral gene expression profiles between IKKε^WT^ and IKKε^KR^ expressing cells using genome-wide quantitative RT-PCR array at 48 hours post-lytic replication showed that IKKε^WT^ substantially increased the overall mRNA levels of KSHV genes in SLK^IKKε KO^-iBAC cells, whereas IKKε^KR^ was unable to rescue this effect (**Fig 3C**). Additionally, the viral DNA copy number was notably elevated in SLK^IKKε KO^-iBAC cells expressing IKKε^WT^ but not IKKε^KR^ (**Fig 3D**). Moreover, analysis of progeny virus from the culture supernatant at 72 hours post-lytic replication revealed that IKKε knockout greatly inhibited KSHV progeny virus production, with only IKKε^WT^, but not IKKε^KR^, restoring progeny virus levels (**Fig 3D**). Furthermore, in BCBL1 cells with IKKε knockout (BCBL1^IKKε KO^), complementation with IKKε^WT^ or IKKε^KR^ via lentiviruses showed that unlike IKKε^WT^, IKKε^KR^ was ineffective in supporting KSHV lytic replication upon TPA treatment, confirming the crucial role of IKKε SUMOylation by ORF45 in KSHV lytic replication and progeny virus production (**Fig 4, A to D**). Together, these results demonstrated that IKKε SUMOylation by ORF45 is essential for the lytic replication and progeny virus production of KSHV.

### IKKε SUMOylation facilitates IKKε-PML interaction and disrupts the formation of PML nuclear bodies

**Fig 5:**
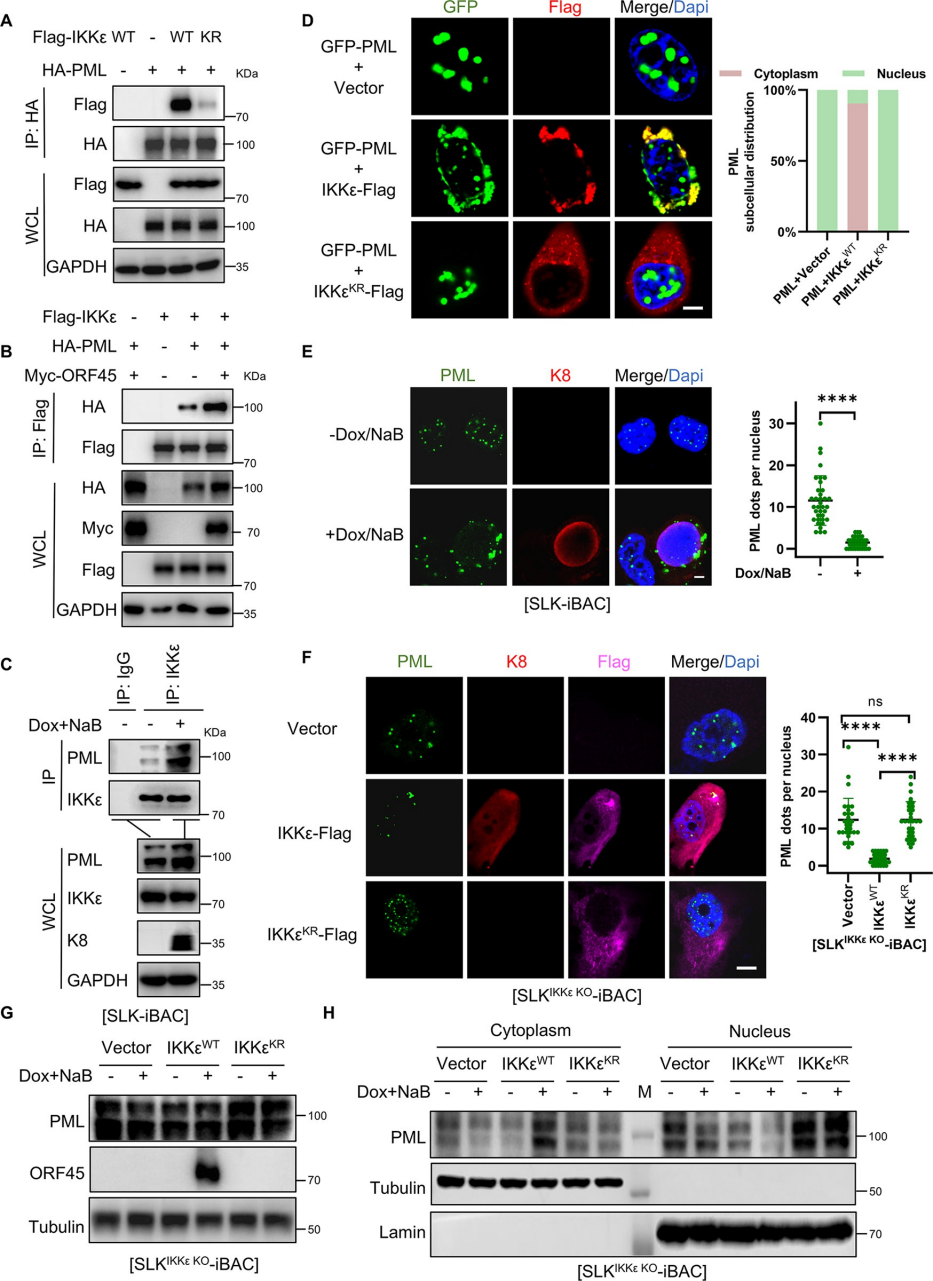
IKKε interacts with PML and disrupts the PML nuclear bodies. (*A*) IKKε interacts with PML. HEK293T cells were co-transfected with indicated plasmids and cell lysates were subjected to IP and IB with indicated antibodies. (*B*) ORF45 promotes IKKε-PML interaction. HEK293T cells were co-transfected with indicated plasmids and cell lysates were subjected to IP and IB with indicated antibodies. (*C*) IKKε-PML interaction was increased during KSHV lytic replication. SLK-iBAC cells were induced with Dox and NaB for lytic replication for 48 h, and the cell lysis were collected for IP and IB with indicated antibodies. (*D*) IKKε expression disrupts PML NB in nucleus. Microscope images and quantification of PML localization in HeLa cells co-transfected with indicated plasmids for 24 h. (*E*) PML NB formation decreases during KSHV lytic replication. Microscope images and quantification of PML NB in SLK-iBAC cells with or without Dox/NaB treatment for 24h. (*F*) SUMOylated IKKε inhibits PML NB formation during KSHV lytic replication. Microscope images and quantification of PML NB in Dox/NaB-induced SLK^IKKε KO^-iBAC cells stably complemented with vector, IKKε, or IKKε^KR^. (*G*) IKKε does not affect PML expression. SLK^IKKε KO^-iBAC stably complemented with vector, IKKε, or IKKε^KR^ with or without Dox/NaB treatment for 48 h, followed by IB with indicated antibody. (*H*) IKKε SUMOylation is required for PML translocation during KSHV lytic replication. Nuclear and cytoplasm fractions were generated from SLK^IKKε KO^-iBAC stably complemented with vector, IKKε, or IKKε^KR^ with or without Dox/NaB treatment for 48 h, followed by IB with indicated antibody. Scale bar, 10 μm in (*D*, *E*, *F*). n = 30; Mean ± SD; ns and ****p < 0.0001 by one-way ANOVA in (*E*, *F*).

A previous study has demonstrated that DNA damage induces IKKε SUMOylation on Lys231, resulting in the co-localization of IKKε with PML in nucleus [37]. It is known that PML or PML-NB components negatively regulate KSHV lytic replication [44,49]. Based on this information, we hypothesized that SUMOylation promotes the association between IKKε and PML during KSHV lytic replication, thereby playing a crucial role in efficient KSHV lytic replication. Indeed, in co-immunoprecipitation assays, wild-type IKKε was found to interact with PML, whereas the SUMOylation-deficient IKKε^KR^ cannot efficiently bound PML (**Fig 5A**). Moreover, both KSHV lytic replication or ORF45 overexpression were observed to significantly enhance the association between IKKε and PML (**Fig 5B and 5C**), suggesting that ORF45-mediated SUMOylation promotes the IKKε-PML association.

PML organizes PML-NBs within the nucleus, which also encompass subunits such as SP100 and DAXX [50]. Co-expression of wild-type IKKε was observed to cause the translocation of PML from the nucleus to a perinuclear region in the cytoplasm where PML co-localized with IKKε, leading to the reduction of PML-NBs in the nucleus (**Fig 5D**). In contrast, the SUMOylation-deficient IKKε^KR^ did not exhibit concentration around the nucleus in the cytoplasm, nor co-localized with PML (**Fig 5D**). Cells expressing IKKε^KR^ showed a similar level of PML-NBs in the nucleus as those expressing the control vector (**Fig 5D**). During KSHV lytic replication, there was a substantial decrease in the number of PML-NBs in wild-type SLK-iBAC cells (**Fig 5E**), while no such reduction was observed in SLK^IKKε KO^-iBAC cells, indicating that KSHV manipulates IKKε to modulate PML-NBs (**S3A Fig**). Consistent with PML-NB formation, the SP100 foci in the nucleus also decreased in wild-type SLK-iBAC cells but remained unchanged in SLK^IKKε KO^-iBAC cells during KSHV lytic replication (**S3B Fig**). Treatment with the IKKε inhibitor BAY-985 reversed the reduction in PML-NBs and SP100 foci levels in SLK-iBAC cells upon KSHV lytic replication (**S3C and S3D Fig**), indicating that the kinase activity of IKKε is essential for modulating PML-NBs. Moreover, stable expression of wild-type IKKε, but not IKKε^KR^, restored PML-NBs and SP100 in the nucleus of SLK^IKKε KO^-iBAC cells during KSHV lytic replication (**Figs 5F** and **S3E**). Notably, the overall expression of PML remained unchanged during KSHV lytic replication (**S3F Fig**), while cell fraction analysis revealed the translocation of PML from the nucleus to the cytoplasm during KSHV lytic replication in SLK-iBAC cells (**S3G Fig**). This translocation was absent in SLK^IKKε KO^-iBAC cells (**S3H and S3I Fig**), but stable expression of wild-type IKKε but not IKKε^KR^ restored PML translocation in these cells (**5F to 5H**). Similarly, KSHV lytic replication enhanced PML-IKKε interaction and disrupted PML NBs in BCBL-1 cells (**S4A and S4B Fig**). And wild-type IKKε but not IKKε^KR^ rescued the disruption of PML NB in BCBL-1I^KKε KO^ cells during KSHV lytic replication (**S4C Fig**). These findings suggest that SUMOylated IKKε inhibits the formation of PML-NBs by promoting the translocation of PML from the nucleus to the cytoplasm.

A previous report demonstrated that knockdown of PML, SP100, or DAXX using siRNA resulted in increased KSHV lytic replication in HFF cells [44]. Consistently, the knockout of PML in SLK-iBAC cells also enhanced KSHV lytic replication. Furthermore, the restoration of PML expression in SLK^PML KO^-iBAC cells rescued KSHV lytic replication, as evidenced by viral mRNA levels (**S4D Fig**), confirming the negative regulatory role of PML in KSHV lytic replication. These findings suggest that KSHV manipulates IKKε to disrupt PML NB formation, which is crucial for efficient KSHV lytic replication (**S4E Fig**).

## Discussion

Post-translational modifications play crucial roles in regulating IKKε activity in various biological processes such as the innate immune response, tumorigenesis, and DNA damage response [29]. As a kinase, IKKε activation relies on the phosphorylation at Ser172 within its activation loop by either itself or TBK1 [51]. IKKε undergoes K63-linked polyubiquitination by cIAP1/cIAP2/TRAF2 ubiquitin E3 ligase complex, a process essential for IKKε-mediated tumorigenesis [52]. Upon viral infection, IKKε can be activated by the unanchored K48-linked polyubiquitin chain generated by TRIM6, leading to downstream STAT1 activation [53]. In the context of DNA damage response, IKKε is SUMOylated at Lys321 by TOPORS SUMO E3 ligase, which is critical for its anti-apoptotic function through NF-κB signaling [37]. Distinct from the DNA damage response, our findings indicate that KSHV lytic replication induces IKKε SUMOylation at both Lys231 and Lys549, which is essential for KSHV lytic cycle. Notably, additional SUMOylation at Lys549 not only facilitates IKKε-PML association but also disrupts the formation of PML NBs, thereby alleviating the inhibitory impact of PML NB on KSHV lytic replication. Our work characterized this first virus encoded SUMO E3 ligase that modifies IKKε to benefit the virus life cycle.

As a core component of PML NBs, PML plays a critical role in limiting herpesvirus infections. Various herpesviruses have developed strategies to evade PML NB-mediated inhibition [54–57]. The HSV-1 immediate-early protein ICP0, a viral ubiquitin E3 ligase, degrades both PML and SP100 during infection, which facilitates efficient HSV-1 replication [58]. HSV-2 ICP27 modulates alternative splicing of PML isoforms, switching from PML-II to PML-V to enhance HSV-2 replication [59]. Varicella-zoster virus (VZV) ORF61, homologous to HSV-1 ICP0, interacts with PML and disrupts PML NBs, contributing VZV pathogenesis in the skin [60]; however, unlike ICP0, VZV ORF61 does not lead to PML degradation [61]. Human cytomegalovirus (HCMV) IE1 protein deSUMOylates PML, resulting in the accumulation of mono-SUMOylated PML and disruption PML NBs [62,63]. EBV immediate-early protein BZLF1 is SUMO1-modified and disrupts PML NBs, facilitating efficient lytic replication of EBV [64]. EBV-encoded kinase BGLF4 also disperses PML NBs, dependent on its SIM and kinase activities [65]. Additionally, EBV EBNA1 hijacks the host kinase CK2 and the deubiquitinase USP7 to disrupt PML NBs, contributing to nasopharyngeal carcinoma [66,67]. Rhesus Macaque Rhadinovirus (RRV)-encoded vIRF R12 disrupts PML NBs and inhibits the transcription of interferon-stimulating genes, aiding viral replication [68]. RRV ORF75 also causes proteasomal degradation of PML and SP100 [69]. KSHV LANA2 increases SUMO2-ubiquitin-modified PML levels and induces PML NB disruption via a proteasome-mediated mechanism, contributing to the malignant progression of PEL [43,70]. KSHV RTA functions as a STUbL, degrading PML and K8 to facilitate an efficient lytic cycle [26]. KSHV ORF75 triggers a relocalization of PML and disperses SP100, antagonizing PML NB-instituted intrinsic immunity [44]. Here, we found that the viral SUMO E3 ligase ORF45 also inhibits PML-mediated restriction of KSHV lytic replication by hijacking the antiviral kinase IKKε. This suggests that KSHV employs multiple viral proteins to deregulate PML throughout its life cycle. Since PML functions as a tumor suppressor [71], disruption of PML NBs by IKKε may also contribute to KSHV-associated tumorigenesis.

As a multi-functional viral protein, KSHV ORF45 employs various linear motifs to modulate the post-translational modifications of both cellular and viral targets [5]. It binds to RSK and ERK through VF-motif and FxFP motif, respectively, thereby maintaining their phosphorylation by preventing dephosphorylation [13,14,72]. Additionally, KSHV ORF45 interacts with USP7 via P/A/E-G-X-S motif, inhibiting the ubiquitination and degradation of ORF33 [10]. Moreover, KSHV ORF45 contains two SIMs and facilitate its interaction with SUMOs, promoting the SUMOylation of its cellular substrates, RSK1 and IKKε [16,17]. Notably, the SUMO E3 ligase activity is not conserved across the gammaherpesvirus family: KSHV ORF45 has two SIMs, RRV ORF45 has only one, while ORF45 from Herpesvirus saimiri (HVS) and EBV lacks any SIMs. This suggests that the ability to mediate SUMO modification has been acquired by KSHV ORF45 during evolution [17]. Given the intricate interplay between SUMOylation and KSHV-encoded proteins, targeting the cellular SUMO modification system may provide a promising strategy for restricting KSHV lytic replication and its associated tumorigenesis.

## Methods

### Cells

HEK293T and HeLa cells were maintained in Dulbecco’s modified Eagle’s medium (DMEM). iSLK.r219, iSLK-BAC16, and iSLK-BAC16-ORF45^stop^ cells were maintained in complete DMEM medium with 400 μg/mL hygromycin, 10 μg/mL puromycin, and 250 μg/mL G418. SLK-iBAC cells were maintained in complete DMEM medium with 400 μg/mL hygromycin [16]. BCBL1 cells were cultured in RPMI 1640 medium. All medium was supplemented with 10% fetal bovine serum (FBS), 2 mM L-glutamine, 100 units/ml penicillin and 100 mg/ml streptomycin at 37°C in a 5% CO2 incubator. Stable cell lines were generated using a standard selection protocol with puromycin (2 μg/mL) or blasticidin (10 μg/mL). Doxycycline (2 μg/mL) and sodium butyrate (1 mM) treatment was used to induce KSHV lytic replication in SLK-iBAC or iSLK-BAC16 cells. TPA (20ng/mL) treatment was used to induce KSHV lytic replication in BCBL1 cells.

### Generation of stable knockout cell lines

sgRNAs were cloned into LentiCRISPR V2-Puro (Addgene #52961). Lentiviruses were generated by co-transfection of LentiCRISPR V2 plasmid carrying corresponding sgRNAs with three packaging plasmids into HEK293T cells and the culture supernatant was collected at 72 h post-transfection. Lentivirus carrying both Cas9 and sgRNA were utilized to infected SLK-iBAC or BCBL1 cells and puromycin selection were performed at 48 h post-infection for at least 2 weeks. The knockout efficiency of the polyclonal cell lines was confirmed by immunoblot with specific antibodies.

### Inhibitor treatment

iSLK.r219 cells were pre-treated with individual kinase inhibitor (10 μM) or DMSO control for 6 h in the complete medium and then treated with doxycycline (2 μg/mL) and sodium butyrate (1 mM) for 24 h to induce the lytic replication. The GFP and RFP images were captured by Keyence Automated High-Resolution microscope (BZ-X800). The cytotoxicity effects of selected inhibitors were determined by Cell Viability Assay with Cell Counting Kit-8 (Beyotime Biotechnology, #C0037).

### Antibodies and chemicals

HRP anti-HA (#901519), HRP anti-Flag (#637311), HRP anti-Myc (#626803) antibodies were purchased from BioLegend. Anti-SUMO1 (#A19121), SUMO2/3 (#A5066), SP100 (#A5851) antibodies were purchased from ABclonal. Anti-IKKε (#D20G4) and anti-TBK1(#3504) antibodies were purchased from Cell Signaling Technology. Anti-PML antibody (#sc-377390) and anti KSHV ORF57 (#sc-135746) were purchased from Santa Cruz. Anti-RTA (ORF50) monoclonal mouse antibody was given by Dr. Ke Lan (Wuhan University, China) [73]. Monoclonal antibodies against ORF45 and polyclonal antibodies against K3 and K8 were described previously [8,74,75]. EZview Red Anti-FLAG M2 Affinity Gel (#F2426), EZview Red Anti-HA M2 Affinity Gel (#E6779), sodium butyrate (#B5887), and TPA (#P1585) were ordered from Sigma. Doxycycline (#S4163) was ordered from Selleck. ClonExpress II One Step Cloning Kit (#C122-01) and HiScript II Q RT SuperMix for qPCR (+gDNA wiper) (#R223-01) were purchased from Vazyme Biotech. Lipofectamine 3000 (#3000015) was purchased from Thermo Fisher Scientific. FuGENE HD Transfection Reagent (#E2311) was purchased from Promega. Small molecule inhibitor library targeting cellular kinases was purchased from TargetMol (#L1600).

### Plasmid constructs

The cDNAs for IKKε and PML were provided by the Core Facility of Basic Medical Sciences, Shanghai Jiao Tong University School of Medicine. RTA, K8, and LANA were amplified from BAC16 by PCR. pCR3.1-ORF45 is originally cloned by Dr. Fanxiu Zhu (Florida State University, USA). pKH3-SUMO1, pKH3-SUMO1^G/A^, and pKH3-SUMO3 were described previously [76]. For cellular expression, IKKε, IKKε mutants, ORF45, RTA, K8, LANA, or PML were cloned into pCDH, pKH3, pCDNA3.1 or pEGFP vectors with indicated tag. For generating stable cell lines, IKKε and IKKε mutants were cloned into pCDH vector with Flag tag. The primer sequences for molecular cloning are included in S2 Table. Guide RNAs (gRNAs) targeting IKKε, TBK1 or PML were cloned into LentiCRISPR V2-Puro (Addgene #52961). Sequences of the sgRNA are: 5’-tccacgttatgatttagacg-3’ for TBK1-sg1; 5’-cgacgcttagtcttagaacc-3’ for TBK1-sg2; 5’-tgcatcgcgacatcaagccg-3’ for IKKε-sg1; 5’-gccccagcaaaaagcgttcg-3’ for IKKε-sg2; 5’-gcggtaccagcgcgactacg-3’ for PML. The gRNA PAM sequences of IKKε and IKKε^K321/549R^ were synonymously mutated to avoid the gene silencing mediated by gRNAs. The point mutations were generated using ClonExpress II One Step Cloning Kit (Vazyme Biotech, #C122-01). All constructs were sequenced using an ABI PRISM 377 automatic DNA sequencer to verify 100% correspondence with the original sequence.

### Immunoprecipitation and immunoblotting

For co-immunoprecipitation, 2×10^6^ HEK293T cells were transfected with 20 μg of plasmid at a confluency of 90% with Lipofectamine 3000 (Thermo Fisher Scientific, #3000015). The cells were washed twice with cold phosphate-buffered saline (PBS) and lysed in a whole cell lysis buffer (WCL) containing (50 mM Tris·HCl [pH 7.4], 150 mM NaCl, 1% NP-40, 1 mM EDTA, 10% glycerol, protease inhibitor cocktail [Roche]) for 20 min on ice at 48h post-transfection. The cell lysates were then centrifuged at 15,000 g for 15 min and the clear supernatants were subjected to immunoprecipitation with anti-Flag M2 agarose resin (Sigma-Aldrich, #F2426) following the manufacturer’s instruction. After 4h incubation at 4°C, the beads were washed for three times with WCL and twice with PBS, and then boiled with the 2×loading buffer for 10 min. The immunoprecipitants were applied to standard immunoblotting analyses with indicated specific antibodies.

Immunoprecipitation in denaturing conditions for detecting SUMOylated protein was described previously [17,76]. Briefly, cells were lysed in SDS lysis buffer made by 1:3 ratio of Buffer I (5% SDS, 0.15 M Tris-HCl pH 6.8, 30% glycerol) and Buffer II (25 mM Tris-HCl pH 8.3, 50 mM NaCl, 0.5% NP-40, 0.5% deoxycolate, 0.1% SDS, 1 mM EDTA) supplemented with protease inhibitors cocktail (Roche), 1 mM DTT and 5 mM N-Ethylmaleimide (NEM, Sigma) and denatured 5 min at 95°C. Cell lysates were then centrifuged at maximum speed for 10 min. Supernatants were either directly resolved by SDS-PAGE or diluted 1:5 in E1A buffer (50 mM Hepes pH 7.5, 250 mM NaCl, 0.1% NP-40, 1 mM EDTA, supplemented with protease inhibitors cocktail, 1 mM DTT and 5 mM NEM) and then immunoprecipitated using anti-Flag M2 agarose resin (Sigma, #F2426). After 4 h incubation at 4°C, the beads were washed for three times with WCL and twice with PBS, and then boiled with the 2 x SDS loading buffer for 10 min. The immunoprecipitants were applied to standard immunoblotting analyses with specific antibodies.

### Immunofluorescence

Indicated cells were seeded into 15 mm glass bottom cell culture dish (NEST, #801002), fixed with 4% paraformaldehyde fix solution (Sangon Biotech, #E672002) for 20 min, permeabilized with Triton X-100 (0.2%) for 30 min, blocked with 1% BSA for 30 min, and immunostained with primary antibodies overnight at 4°C, followed by a fluorescent secondary antibody for 1 h at room temperature. DAPI (Thermo Fisher Scientific, #62248) was used to mark nuclei. Primary antibodies used in this study included rabbit anti-Flag (Cell Signaling Technology, #14793, 1:400), mouse anti-Flag (Cell Signaling Technology, #9A3, 1:400), rabbit anti-SP100 (ABclonal, #A5851, 1:100), and mouse anti-PML (Santa Cruz, #sc-377390, 1:50). Confocal images were captured with Leica SP8 confocal microscope and analyzed with Leica Application Suite X and ImageJ. The number of PML and SP100 dots per cells was counted manually in three independent experiments.

### Nuclear and cytoplasmic extraction

Nuclear and cytoplasmic fractions were separated with Nuclear and Cytoplasmic Protein Extraction Kit (Beyotime, #P0028) according to the manufacturer’s instructions. Briefly, indicated cells were collected and pelleted by centrifugation at 500 g for 5 min. After washing with cold PBS, 200 μL Cytoplasmic Extraction Reagent A was added to the cell pellets, and the mixture was vortexed gently to suspend the cell pellets. The mixture was kept on ice for 10 min, and 10μL ice-cold Cytoplasmic Extraction Reagent B was added to the mixture. After vortexing for 5 s, the mixture was centrifuged for 10 min at 16,000 g. The supernatant (cytoplasmic extract) was transferred to a clean prechilled tube, and the insoluble (pellet) fraction was suspended in 50 μL ice-cold Nuclear Extraction Reagent. The pellet suspended in Nuclear Extraction Reagent was vortexed every 10 min for a total of 40 min and then centrifuged at 16,000 g for 10 min. The supernatant (nuclear extract) fraction was immediately transferred to a clean prechilled tube. The clear nuclear or cytoplasmic supernatants were subjected to immunoblotting to evaluate PML translocation.

### *In vitro* SUMOylation assay

*In vitro* SUMOylation assay was performed as described previously [16]. SUMO1 (#K-700), SAE1/UBA2 (#E1-315), and ubc9 recombinant proteins (#E2-645), SUMO conjugation reaction buffer (#SK-15), and ATP/Mg (#SK-15) were purchased from Boston Biochem. Flag-IKKε, HA-ORF45, or HA-ORF45^SIM^ were purified from transfected HEK293T cells by affinity purification via anti-Flag or anti-HA M2 affinity Gel. Briefly, Flag-tagged IKKε and HA-tagged ORF45 was purified from transfected HEK293T cells by affinity purification via anti-Flag M2 affinity Gel (Sigma, #F2426) or anti-HA M2 affinity Gel (Sigma, #E6779). Similar with co-immunoprecipitation, 8×10^6^ HEK293T cells were transfected with 20 μg of plasmid at a confluency of 90% with Lipofectamine 3000 (Thermo Fisher Scientific, #3000015). The cells were washed twice with cold phosphate-buffered saline (PBS) and lysed in a whole cell lysis buffer (WCL) containing (50 mM Tris·HCl [pH 7.4], 150 mM NaCl, 1% NP-40, 1 mM EDTA, 10% glycerol, protease inhibitor cocktail [Roche]) for 20 min on ice at 48h post-transfection. The cell lysates were then centrifuged at 15,000 g for 15 min and the clear supernatants were subjected to anti-Flag or anti-HA M2 agarose resin following the manufacturer’s instruction. After 4-6h incubation at 4°C, the beads were washed for three times with WCL and twice with PBS, then resuspended with 100 μL Flag or HA peptide eluent (150 μg/mL) and incubation at 4°C for 2h. Centrifuge at 9000 rpm for 30 seconds at 4°C and carefully transfer the supernatant to a new tube. The protein concentration was determined by BCA Protein Assay and the purified proteins store at -80°C. 1 μg purified IKKε substrate was mixed with 10 μM SUMO1, 250 nM SAE1/UBA2, 1 μM ubc9, 1 μg ORF45 or ORF45^SIM^, 1 x ATP/Mg, and 1 x SUMO reaction buffer in 20 μL reaction system for 3 h at 30°C and stopped by adding stop buffer (Boston Biochem, #SK-15). The reaction mixture was subjected to standard immunoblotting analyses to detect the SUMOylation of IKKε.

### RNA purification and RT-qPCR

Cells were seeded in 12-well plate and over 10^6^ cells were collected for RNA extraction. Total RNA was extracted from cells with TRIzol reagent (Sigma) according to the manufacturer’s protocol. Briefly, 1 μg of total RNA was reverse transcribed by HiScript II Q RT SuperMix for qPCR (+gDNA wiper) (Vazyme Biotech, #R223-01) and the cDNA was quantified by SYBR green (Vazyme, #Q312-02) based qPCR using gene specific primers. Experiments run on the QuantStudio 7Flex System (Thermo Fisher), and qPCR program sets as follows: 1. Pre-denaturation stage: 95°C, 10min (1.6°C/s); 2. PCR stage: 95°C, 15s (1.6°C/s), 60°C, 1min (1.6°C/s), 40 cycles; 3. Melt curve stage: 95°C, 15 s (1.6°C/s), 60°C, 1 min (1.6°C/s), 95°C, 15 s (0.05°C/s). The relative level of gene expression was calculated by the fold change (2^-ΔΔCt^) between the experimental samples and the control, while GAPDH was used for normalization. The RT-qPCR graphs represent the average of at least three independent experiments. The sequences of the primers used in RT-qPCR have been described previously [16,17], and listed in the supplementary table (S3 Table).

### Quantification of intracellular and extracellular virion genomic DNA

SLK-iBAC and its derived cells were induced with doxycycline (2 μg/mL) and sodium butyrate (1 mM) for KSHV reactivation, BCBL1 and its derived cells were treated with TPA (20 ng/mL) for KSHV lytic replication. Total intracellular DNA was purified with a cell DNA isolation Kit (Vazyme Biotech, #DC102-01) according to the manufacturer’s instructions. The viral DNA was measured by qPCR using primers for LANA and normalized to GAPDH. Extracellular supernatant viral DNA was purified with a viral DNA isolation Kit (Vazyme Biotech, #RC311). The KSHV genomic DNA in virions was measured by qPCR with KSHV-specific primers ORF11. Viral DNA copy numbers were calculated with external standards of known concentrations of serially diluted BAC16 DNA ranging from 1 to 10^7^ genome copies per reaction.

### Quantification and statistical analysis

All data were expressed as Mean ± s.d., unless otherwise noted. For parametric analysis, the F test was used to determine the equality of variances between the groups compared; statistical significance across two groups was tested by two-tailed unpaired Student’s t-test; one-way analysis of variance (ANOVA) followed by Bonferroni’s *post hoc* test were used to determine statistically significant differences between multiple groups. *P*-values of less than 0.05 were considered significant.

## Supporting information

S1 Fig IKKε but not TBK1 affects KSHV lytic replication. (*A*) The effects of indicated inhibitors for KSHV lytic replication. The iSLK.r219 cells were pre-treated with 10 μM indicated inhibitors for 6 hours, and induced by Dox/NaB for 24 hours. The GFP and RFP images were captured by Keyence Automated High-Resolution microscope (BZ-X800). Scale bar, 100 μm. (*B*) Validation of the effects of indicated inhibitors on viral lytic gene expression by qRT-PCR in SLK-iBAC cells. SLK-iBAC cells were pre-treated with indicated inhibitors (10 μM) or DMSO for 6 h, followed by induction with Dox/NaB for 48 h. Total RNA was extracted and the indicated viral genes expression levels were analyzed by qRT-PCR. The bar graph shows the relative expression levels of RTA, K8, or K3 with indicated inhibitors relative to DMSO treatment. (*C*-*D*) TBK1 does not affect KSHV lytic replication. SLK-iBAC and SLK^TBK1 KO^-iBAC (2 clones) were treated with Dox/NaB for 48 h to induce KSHV lytic replication. Cell lysates were collected and subjected to IB with indicated antibodies (*C*). Total DNA was isolated from the culture supernatant of indicated cells and viral genomic DNA was quantified by qPCR (*D*). Data represent the means of three independent experiments in (*B*, *D*); Mean ± SD; ns by one-way ANOVA in (*D*).
(TIFF)

S2 Fig IKKε is SUMOylated by ORF45 on Lys231 and Lys549. *(A*) KSHV ORF45 promotes IKKε SUMOylation. HEK293T cells were co-transfected with indicated plasmids and cell lysates were subjected to IP under denature condition and IB with indicated antibodies. (*B*) aa 300–500 and aa 500–716 of IKKε interact with ORF45. HEK293T cells were co-transfected with indicated plasmids and cell lysates were subjected to IP and IB with indicated antibodies. (*C*) ORF45 is required for IKKε SUMOylation during KSHV lytic replication. iSLK-BAC16 and iSLK-BAC16-ORF45^stop^ cells were induced with Dox and NaB for lytic replication. Cell lysates were harvested at 48 h post-induction and subjected to IP under denature condition and IB with indicated antibody. (*D*) Lys231 and Lys549 are the SUMOylation sites of IKKε by ORF45. HEK293T cells were co-transfected with indicated plasmids and cell lysates were subjected to IP under denature condition and IB with indicated antibodies.
(TIFF)

S3 Fig IKKε disrupts the PML nuclear bodies during KSHV lytic replication. (*A*-*B*) IKKε inhibits PML-NB and SP100 foci formation during KSHV lytic replication. Microscope images and quantification of PML NB (*A*) or SP100 foci (*B*) in SLK-iBAC or SLK^IKKε KO^-iBAC cells with or without Dox/NaB treatment for 48 h. (*C-D*) BAY985 blocks PML-NB and SP100 foci formation during KSHV lytic replication. Microscope images and quantification of PML NB (*C*) or SP100 foci (*D*) in SLK-iBAC cells with or without BAY985 treatment for 6 h, followed by Dox/NaB treatment for 48 h. (*E*) SUMOylated IKKε inhibits SP100 foci in nucleus during KSHV lytic replication. Microscope images and quantification of SP100 foci in Dox/NaB-induced SLK^IKKε KO^-iBAC cells stably complemented with vector, IKKε, or IKKε^KR^. (*F*) IKKε does not affect the overall expression of PML during KSHV lytic replication. SLK-iBAC cells or SLK^IKKε KO^-iBAC cells stably complemented with vector, IKKε, or IKKε^KR^ were treated with or without Dox/NaB for 48 h. The whole cell lysates were collected and subjected to IB with indicated antibodies. (*G*) Nuclear and cytoplasm fractions were generated from SLK-iBAC with or without Dox/NaB treatment for 48 h, followed by IB with indicated antibodies. (*H-J*) IKKε SUMOylation is required for PML translocation during KSHV lytic replication. Whole cell lysates (*H*), nuclear and cytoplasm fractions (*I*) were generated from SLK-iBAC or SLK^IKKε KO^-iBAC with or without Dox/NaB treatment for 48 h, followed by IB with indicated antibody. PML localization was quantified by band intensity with ImageJ (*J*). Scale bar, 10 μm in (*A* to *E*); n = 6 (*A*, *B*), n = 20 (*C*, *D*), n = 16 (*E*). Mean ± SD; ns, **p < 0.01, ***p < 0.001, and ****p < 0.0001 by one-way ANOVA in (*A* to *E*).
(TIF)

S4 Fig KSHV lytic replication enhanced PML-IKKε interaction and disrupted PML NBs in BCBL-1 cells. (*A*) IKKε-PML interaction was increased during KSHV lytic replication. BCBL1 cells were induced with TPA for lytic replication for 72 h, and the cell lysis were collected for IP and IB with indicated antibodies. (*B*) PML NB formation decreases during KSHV lytic replication. Microscope images and quantification of PML NB in TPA-induced BCBL1 cells. (*C*) SUMOylated IKKε inhibits PML NB formation during KSHV lytic replication. Microscope images and quantification of PML NB in TPA-induced BCBL1^IKKε KO^ cells stably complemented with vector, IKKε, or IKKε^KR^. (*D*) PML inhibits KSHV lytic replication. SLK-iBAC and SLK^PML KO^-iBAC cells were transiently transfected with plasmids expressing PML or vector control for 24 h, followed by Dox/NaB treatment for 48 h. The indicated viral gene expressions were quantified by qRT-PCR at 48 h post-treatment. (E) Graphical model for the role of IKKε SUMOylation in KSHV lytic replication. Scale bar, 10 μm in (*B*, *C*). n = 30 (*B*); n = 20 (*C*); n = 3 (*D*). Mean ± SD; ns, **p < 0.01, ***p < 0.001, and ****p < 0.0001 by one-way ANOVA in (*B* to *D*).
(TIF)

S1 Table Original data for kinase inhibitor screening on KSHV lytic replication. (PDF)

S2 Table Primer sequence for cloning. (XLSX)

S3 Table Primer sequence for KSHV RT-qPCR array. (XLSX)
