# Supplementary figures and images for: KSHV hijacks the antiviral kinase IKKε to initiate lytic replication
Source: PLoS Pathog. 2025 Jan 17;21(1):e1012856. doi: 10.1371/journal.ppat.1012856 (PMC11781660; doi:10.1371/journal.ppat.1012856)

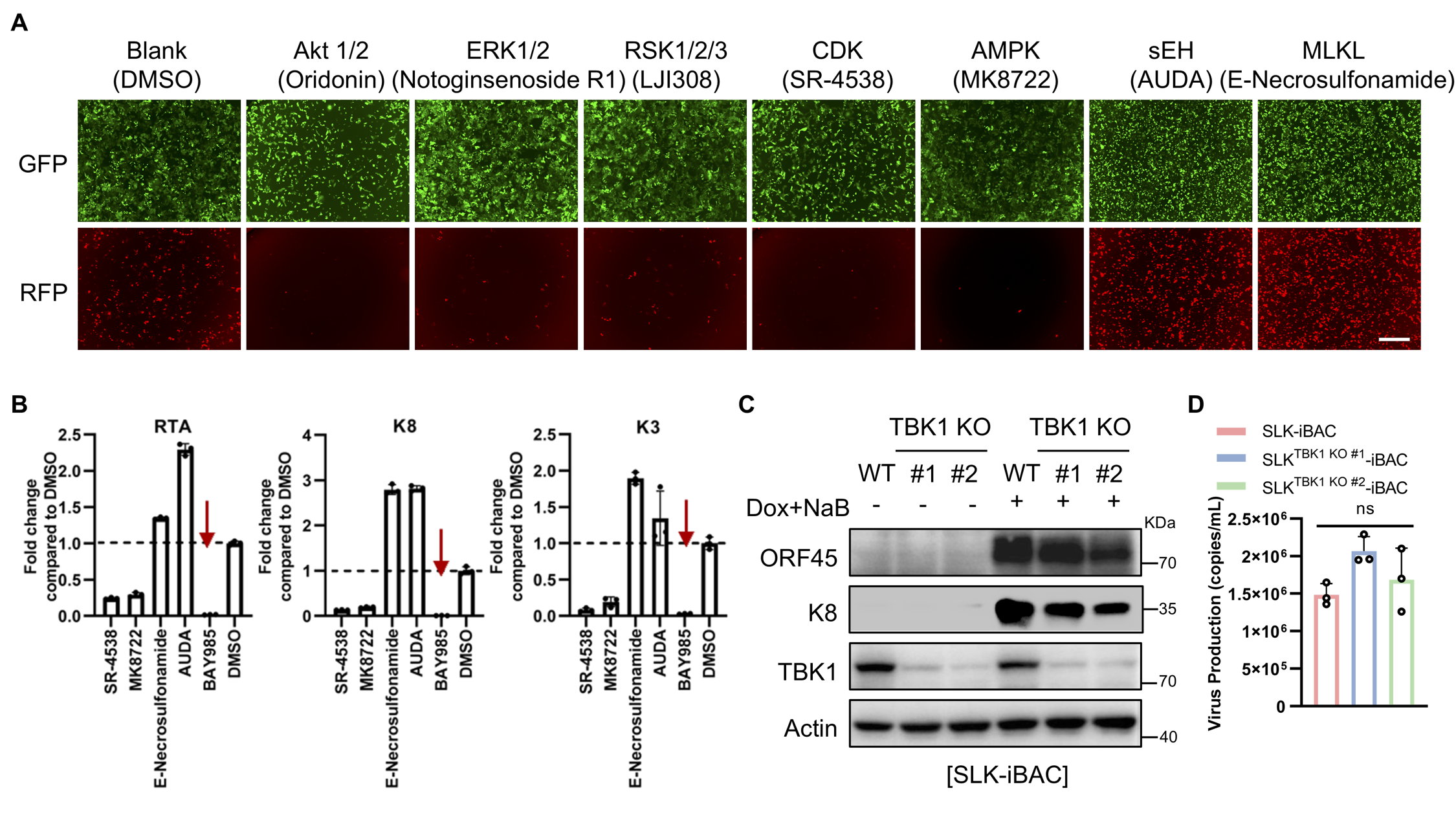

Supplement: S1 Fig — (A) The effects of indicated inhibitors for KSHV lytic replication. The iSLK.r219 cells were pre-treated with 10 μM indicated inhibitors for 6 hours, and induced by Dox/NaB for 24 hours. The GFP and RFP images were captured by Keyence Automated High-Resolution microscope (BZ-X800). Scale bar, 100 μm. (B) Validation of the effects of indicated inhibitors on viral lytic gene expression by qRT-PCR in SLK-iBAC cells. SLK-iBAC cells were pre-treated with indicated inhibitors (10 μM) or DMSO for 6 h, followed by induction with Dox/NaB for 48 h. Total RNA was extracted and the indicated viral genes expression levels were analyzed by qRT-PCR. The bar graph shows the relative expression levels of RTA, K8, or K3 with indicated inhibitors relative to DMSO treatment. (C-D) TBK1 does not affect KSHV lytic replication. SLK-iBAC and SLKTBK1 KO-iBAC (2 clones) were treated with Dox/NaB for 48 h to induce KSHV lytic replication. Cell lysates were collected and subjected to IB with indicated antibodies (C). Total DNA was isolated from the culture supernatant of indicated cells and viral genomic DNA was quantified by qPCR (D). Data represent the means of three independent experiments in (B, D); Mean ± SD; ns by one-way ANOVA in (D). (TIFF) [file ppat.1012856.s001.tiff]

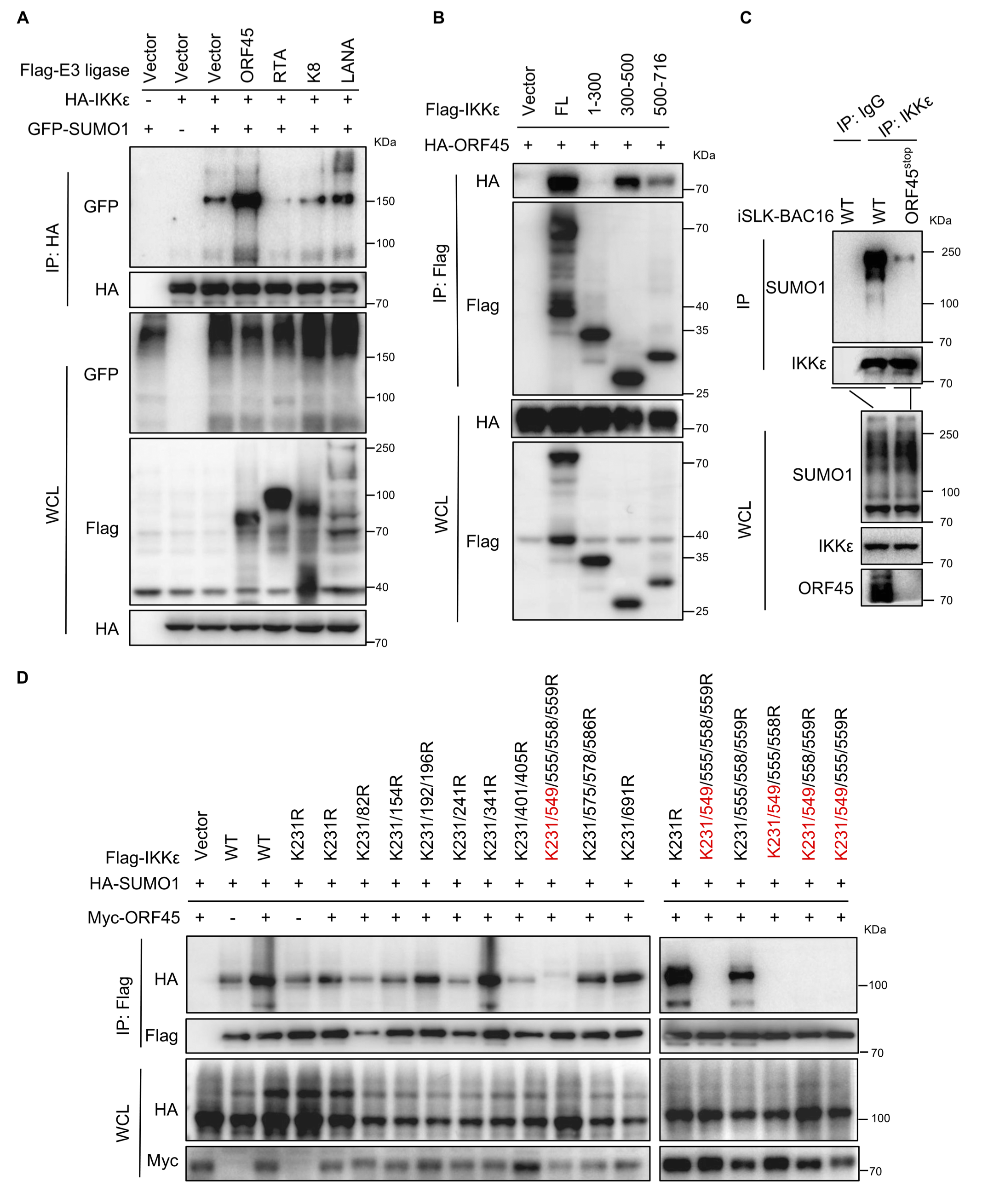

Supplement: S2 Fig — (A) KSHV ORF45 promotes IKKε SUMOylation. HEK293T cells were co-transfected with indicated plasmids and cell lysates were subjected to IP under denature condition and IB with indicated antibodies. (B) aa 300–500 and aa 500–716 of IKKε interact with ORF45. HEK293T cells were co-transfected with indicated plasmids and cell lysates were subjected to IP and IB with indicated antibodies. (C) ORF45 is required for IKKε SUMOylation during KSHV lytic replication. iSLK-BAC16 and iSLK-BAC16-ORF45stop cells were induced with Dox and NaB for lytic replication. Cell lysates were harvested at 48 h post-induction and subjected to IP under denature condition and IB with indicated antibody. (D) Lys231 and Lys549 are the SUMOylation sites of IKKε by ORF45. HEK293T cells were co-transfected with indicated plasmids and cell lysates were subjected to IP under denature condition and IB with indicated antibodies. (TIFF) [file ppat.1012856.s002.tiff]
