# Supplementary material for: KSHV hijacks the antiviral kinase IKKε to initiate lytic replication
Source: PLoS Pathog. 2025 Jan 17;21(1):e1012856. doi: 10.1371/journal.ppat.1012856 (PMC11781660; doi:10.1371/journal.ppat.1012856)
Supplement: S1 Table — (PDF) [file ppat.1012856.s005.pdf]

Targets	Ratio of RFP+ cells (Treated/DMSO)	Molecule Name
Adenosine kinase	0.2547	ABT-702 dihydrochloride
choline kinase alpha 1 (ChoKα1)	0.3745	EB-3D
mAChR	0.4944	Scopoletin
ACK1	0.5693	AIM100
Akt1,Akt2;AKT2	0.0599	Oridonin
Akt1; Akt2; Akt3	0.2097	AKT inhibitor VIII
ALK5	0.0599	R-268712
ALK5	0.1723	GW 788388
ALK5; ALK4; ALK7	0.6067	SB 431542
ALK4; ALK5; ALK7	0.3970	ALK5 Inhibitor IV
ALK4; ALK5	0.6667	SM 16
ALK4; ALK5	1.3184	SB505124
ALK	0.0225	ALK inhibitor 2
ALK	0.1648	CEP28122
ALK2	0.4869	DMH1
ALK2	0.8614	LDN214117
ALK1; ALK2; ALK3; ALK4; ALK5	0.7191	LDN212854
ALK1; ALK2; ALK3; ALK4; ALK6	0.9064	K 02288
ALK1; ALK2; ALK3; ALK6	0.7790	ML347
AMPK	0.0097	MK3903
AMPK	0.0150	MK8722
AMPK	0.1354	EX229
NUAK1; NUAK2	0.2996	WZ4003
NUAK1; NUAK2	0.8914	HTH-01-015
Antibacterial;Antiviral	0.9438	Esculin
Antibiotic	0.8895	Dihydrostreptomycin sulfate
antioxidant	0.9513	Isorhamnetin 3-O-neohesperidin
antioxidant	1.4906	Oroxin B
IRAK-4	0.0870	IRAK inhibitor 6
Cdc20	1.4607	Apcin
ASK1	0.8218	GS444217
ATM	0.0375	CP466722
ATM; ATR	0.1049	CGK 733
MRN	1.1086	Mirin
non-muscle myosin II ATPases	0.6217	(-)-Blebbistatin
Aurora B (human); TbAUK1	0.1348	Hesperadin
Aurora A	0.6142	Aurora Kinase Inhibitor III
Serine/threonine-protein kinase Aurora-A	1.1215	TLC1218-1281
Aurora A; Aurora B; Aurora C	0.9288	CCT137690
Aurora A; Aurora B	1.7453	MK8745
Autophagy-ULK1	0.0300	BL-918
Autophagy-ULK1; ULK2	0.2127	MRT68921 HCl
Autophagy	0.0899	Usnic Acid
mCLK1; mCLK2; mCLK4	0.9888	TG003
Bcr-Abl1	0.4419	GMB-475
Abl	0.5317	CZC-8004
Tyrosine-protein kinase ABL	0.8895	TLC9995-0188
Tyrosine-protein kinase ABL	0.8991	TLC0417-1975
Tyrosine-protein kinase ABL	0.9281	TLC0417-1893
Tyrosine-protein kinase ABL	0.9571	TLC0509-1188
Tyrosine-protein kinase ABL	1.0151	TLC0417-1984
Tyrosine-protein kinase ABL	1.0538	TLC0417-1904
Tyrosine-protein kinase ABL	1.0635	TLC0344-0932
Tyrosine-protein kinase ABL	1.2278	TLC0417-1895
Tyrosine-protein kinase ABL	1.6339	Y040-3432
BMI-1	1.1536	XMD8-92
BTK	0.1124	CGI1746
BTK	0.1124	CNX774
Bmx	1.4457	CHMFL-BMX-078
NCX	0.6517	SEA0400

CaMKII	1.3052	CaM kinase II inhibitor TFA salt
CaMKII	1.0861	KN-93 Phosphate
CaMKⅤ; CaMKII	0.5543	KN62
CaMK	0.6442	KN-92 phosphate
CaMK	1.5206	KN93
CaMKP	0.9963	CaMKP Inhibitor
CaM-KKα; CaM-KKβ	1.1610	STO609
CaM-KKα; CaM-KKβ	1.5056	STO-609 acetate
CK2	0.0449	DMAT
CK2	0.0075	Emodin
CK1ε	0.2247	PF4800567
CK1ε	0.4120	PF670462
Casein kinase I delta	0.8701	TLC2014-0078
Casein kinase I delta	1.2955	Y030-2976
CDK	0.0375	SR-4835
CDK7	0.0075	LDC-4297 HCl (1453834-21-3(free base))
CDK8	0.0075	MSC2530818
CDC7	0.0097	LY3143921 hydrate
Cdc7	0.0300	LY-3177833
CLK	0.0483	SRI-29329
CLK2;TTK	0.0749	CC671
P18INK	1.1236	NSC23005
p18INK4C	1.2060	NSC23005 Sodium
CSF1R	0.4254	cFMS Receptor Inhibitor II
c-Fms	0.9588	GW 2580
CHK1	1.4082	SAR-020106
Kit	0.4641	AZD3229
c-Kit	0.6367	ISCK03
MET RTK	0.9185	SAR125884 hydrochlorid (1116743-46-4(free base))
EGFR,c-Met	0.9281	Norcantharidin
c-Met	1.0487	BAY-474
c-Met	1.3933	Capmatinib xHCl
c-Met	1.4382	c-Met inhibitor 1
HGF	1.2665	Dihexa
MYC	0.4944	MYCi361
MYC	0.8614	MYCi975
c-Myc-MAX	0.2697	10058-F4
COX	0.8689	Indoprofen
RET	0.0000	BBT594
PDHK1; PDHK2	0.3895	AZD7545
DDR1/2	0.3745	VU6015929
DDR1	0.9185	DDR1-IN-2
DDR1; DDR2	1.0037	Ddr1-In-1
DNA replication	0.3670	HAMNO
DNA-PK	0.7341	NU7441
DNA-PK	0.6592	Compound 401
HDR	1.1985	YU238259
DYRK	0.0524	ID8
DYRK1A	0.0677	TLC0348-1309
DYRK1A	0.1547	TLC1345-0274
DYRK1A; DYRK1B; DYRK2	0.1124	AZ191
Ubiquitin-activating Enzyme E1	0.5993	PYR41
EGFR	0.0449	Erlotinib hydrochloride
hERG;EGFR	0.0974	AG1557
EGFR; EGFR	0.0599	PD153035 hydrochloride
EGFR	0.1648	Avitinib maleate
EGFR (mutant)	0.1723	CNX2006
HER2/ErbB2	0.4419	CP724714

EGFR; HER2/ErbB2	0.7191	Lapatinib ditosylate monohydrate
EGFR;ErbB2	0.9213	Tyrphostin AG 528
EGFR; HER2/ErbB2	0.9438	Canertinib dihydrochloride
EGFR/ErbB1	0.9513	EBE-A22
EGFR/VEGFR2	0.4351	ZD-4190
hERG Potassium Channel	0.7734	E-4031
Ephrin Receptor	0.6381	Ehp-inhibitor-1
Ephrin Receptor	0.7831	Ehp-inhibitor-2
BRD2; BRD3; BRD4	2.3745	I-BET151
ERK1; ERK2	0.1049	Notoginsenoside R1
ERK1; ERK2	0.0524	SCH772984
ERK1/2	0.2247	ASTX029
MAP kinase ERK2	0.2224	4593-2847
ERK5	1.4405	ERK5-IN-2
ERK5	1.7828	ERK5-IN-1
ERK5	1.6404	XMD17109
ERK5	1.1795	AX-15836
PERK	0.7566	ISRIB
PERK	1.5805	ISRIB (trans-isomer)
CERK	1.1536	NVP 231
ERK;MEK	0.3820	BIX 02189
ERS	0.6142	Astragaloside IV
PTK2/FAK	0.6367	BI-4464
FAK	1.7154	Y15
FAK	0.6592	PF 573228
FAK	0.7416	(+)-Fangchinoline
FAK	2.2996	Corosolic acid
FGFR3	0.4447	Dovitinib lactate
FGFR	0.6816	Formononetin
FGFR1	0.4045	PD166866
FGFR1	0.8165	SSR128129E
FGFR1; FGFR2; FGFR3; FGFR4	0.5693	FIIN2
FGFR1; FGFR2; FGFR3; FGFR4	1.2809	FIIN3
FGFR1; FGFR2; FGFR3; FGFR4	1.8577	BLU9931
FLT3	0.2772	BPR1J-097 hydrochloride (1327167-19-0(free base))
Tyrosine-protein kinase receptor FLT3	0.7058	TLC1358-0295
Tyrosine-protein kinase receptor FLT3	0.9475	TLC0016-0634
Tyrosine-protein kinase receptor FLT3	0.9475	TLC0328-0539
Tyrosine-protein kinase receptor FLT3	0.9861	TLC0016-0682
Tyrosine-protein kinase receptor FLT3	1.0248	TLC0098-0102
Tyrosine-protein kinase receptor FLT3	1.1118	TLC0863-0553
Tyrosine-protein kinase receptor FLT3	1.1408	TLC0016-0637
Tyrosine-protein kinase receptor FLT3	1.1988	TLC0825-0033
FLT3	1.2285	5'-Fluoroindirubinoxime
Tyrosine-protein kinase receptor FLT3	1.2568	TLC0098-0101
Tyrosine-protein kinase receptor FLT3	1.3052	TLC0806-0389
Tyrosine-protein kinase receptor FLT3	1.4019	TLC0126-0221
Tyrosine-protein kinase receptor FLT3	1.4115	TLC0016-0657
Tyrosine-protein kinase receptor FLT3	1.4792	TLC0016-0636
Tyrosine-protein kinase receptor FLT3	1.5566	TLC0016-0676
Tyrosine-protein kinase receptor FLT3	1.5856	TLC0016-0677
FLT3	0.3371	FLT3-IN-2
FLT3	0.6367	BPR1J097
FLT3	1.0248	TCS 359
Free radical scavengers	1.1685	2,2,6,6-Tetramethylpiperidinooxy
GLP-1	0.8464	Shanzhiside methyl ester
6-phospho-1-fructokinase	0.4447	Y050-2136
6-phospho-1-fructokinase	0.5607	E854-2666
6-phospho-1-fructokinase	0.5704	E591-0194

6-phospho-1-fructokinase	0.5994	TLC0451-2187
6-phospho-1-fructokinase	0.6768	4100-0150
6-phospho-1-fructokinase	0.7734	K261-2105
6-phospho-1-fructokinase	0.8218	4606-2041
6-phospho-1-fructokinase	0.8218	TLC2592-0053
6-phospho-1-fructokinase	0.8508	5408-2526
6-phospho-1-fructokinase	0.8895	K781-4683
6-phospho-1-fructokinase	0.8991	E715-0343
6-phospho-1-fructokinase	0.9378	Y030-6111
6-phospho-1-fructokinase	0.9378	TLC9995-0403
6-phospho-1-fructokinase	0.9861	E203-0583
6-phospho-1-fructokinase	1.0151	K263-0647
6-phospho-1-fructokinase	1.0248	5867-4109
6-phospho-1-fructokinase	1.0345	E138-0961
6-phospho-1-fructokinase	1.0345	D264-0224
6-phospho-1-fructokinase	1.0787	8015-4922
6-phospho-1-fructokinase	1.1022	6058-0043
6-phospho-1-fructokinase	1.1022	Y030-7081
6-phospho-1-fructokinase	1.1610	C365-0181
6-phospho-1-fructokinase	1.1892	6186-3464
6-phospho-1-fructokinase	1.1892	Y020-7439
6-phospho-1-fructokinase	1.2375	TLC2017-0066
6-phospho-1-fructokinase	1.2472	TLC2660-0074
6-phospho-1-fructokinase	1.2734	C327-0112
6-phospho-1-fructokinase	1.2734	8012-3546
6-phospho-1-fructokinase	1.3149	Y030-8651
6-phospho-1-fructokinase	1.3633	8388-0772
6-phospho-1-fructokinase	1.3729	TLC3145-0274
6-phospho-1-fructokinase	1.4532	8012-4007
6-phospho-1-fructokinase	1.5581	8006-6156
6-phospho-1-fructokinase	1.6105	8005-9275
6-phospho-1-fructokinase	1.6629	8013-3111
Glucokinase	1.2210	AM2394
Glucokinase	1.0487	MK-0941 free base
β-D-galactosidase; β-D-glucosidase	0.6574	Tryptophan, N-indol-3-ylacetyl-(6CI)
GL1 synthase	1.0787	Genz-123346 free base
PFKFB3	2.0974	(E)-3PO
GPR120; GPR40(FFA1)	0.3670	GW 9508
G-protein coupled receptor kinase 2	0.7154	K978-0819
GRK2	1.2665	1-(2,3-DIHYDRO-BENZO[1,4]DIOXIN-6-YL)-5-OXO-PYRROLIDINE-3-CARBOXYLIC ACID
G-protein coupled receptor kinase 2	1.3632	TLC0613-0132
Glycogen synthase kinase-3 beta	0.0580	TLC2771-0100
Glycogen synthase kinase-3 beta	0.1063	TLC0919-0843
Glycogen synthase kinase-3 beta	0.1063	TLC2771-0099
GSK-3β; GSK-3β	0.0749	AR-A014418
GSK-3	0.9438	GS87
GSK-3	1.2665	5-Bromoindole
GSK-3	1.6255	AZD2858
GSK-3α; GSK-3β	2.2697	CHIR99021
GSK-3α; GSK-3β	1.8352	BIO-acetoxime
GSK-3α; GSK-3β	2.3596	SB216763
GSK-3;PDE7	2.3977	VP3.15 dihydrobromide
GTPase Ral	1.3109	BQU57
HER2; JAK2	2.2846	Mollugin
Putative hexokinase HKDC1	0.7444	TLC2005-0121

Putative hexokinase HKDC1	1.1988	TLC0741-0005
Putative hexokinase HKDC1	1.9433	TLC0723-0024
Histone Demethylase;Mitochondrial Metabolism	1.1602	L-2-Hydroxyglutaric acid disodium
HIV-1, A012 isolate;S-adenosylhomocysteine hydrolase	0.2997	3-Deazaadenosine hydrochloride
IGF-1R	0.1948	PQ401 hydrochloride (196868-63-0(free base))
IGF-1R; Insulin Receptor	0.1273	AG1024
IL-12; IL-23	1.4007	Apilimod mesylate
interleukin-1	0.4569	RP-54745
gp130	0.6574	RCGD423
IRE1α	0.0000	6-Bromo-2-hydroxy-3-methoxybenzaldehyde
IKK1;IKK2	0.0524	BMS-345541 hydrochloride
IKK2	0.1798	TPCA1
IKK2	0.2322	LY2409881 trihydrochloride
TBK1;TBK1;IKKε	0.0199	BAY-985
TBK1;IKKε	0.0648	TBK1/IKKε-IN-2
TBK1; IKKϵ	0.2146	TBK1/IKKε-IN-5
IKKα; IKKβ	0.5027	INH14
IKKβ	1.2665	2-AMINO-5-PHENYL-THIOPHENE-3-CARBOXYLIC
IKCa1 (KCa3.1)	1.4157	TRAM34
JAK3	0.2610	FM381
JAK3	0.4494	WHI-P97 HCl 211555-05-4(free base)
JAK3	0.8315	JAK3-IN-6
JAK3; JAK3	0.5918	WHI-P131
JAK2	0.2022	CEP33779
JAKs	0.0375	Oclacitinib
WDR5	0.9064	WDR5-0103
WDR5	1.3858	OICR9429
TGM2 , JAK3 , EGFR , JAK1	1.0861	ZM 39923 HCl
JNK1;JNK2;JNK3	0.4120	JNK Inhibitor VIII
JNK3;JNK1;JNK2	1.0562	IQ3
c-Jun N-terminal kinase 1	0.7638	TLC1358-0352
c-Jun N-terminal kinase 1	0.8218	TLC2976-0823
c-Jun N-terminal kinase 1	0.8218	TLC2995-0219
c-Jun N-terminal kinase 1	0.9861	TLC2726-0893
c-Jun N-terminal kinase 1	1.0635	TLC2300-0358
c-Jun N-terminal kinase 1	1.3535	TLC1358-1003
c-Jun N-terminal kinase 1	1.3729	TLC1358-0264
JNK1	0.2697	DB07268
JNK2; JNK3	1.6180	SC202671
c-Jun N-terminal kinase 3	0.7928	K081-0174
c-Jun N-terminal kinase 3	1.0055	TLC1284-0835
c-Jun N-terminal kinase 3	1.1505	TLC3407-3739
c-Jun N-terminal kinase 3	1.2859	TLC0016-0395
c-Jun N-terminal kinase 3	1.3149	TLC0016-0399
Mps1	0.6292	MPI0479605
Mps1	0.8090	AZ 3146
BLT2	0.8090	LY 255283
LIMK	0.3221	TH 257
LIM domain kinase 1	1.1215	TLC0777-0306
LIM domain kinase 1	1.2360	8005-8383
LIMK1;LIMK2	1.2434	BMS3
LIMK1;LIMK2	1.9176	BMS5
LIMK2	1.3109	T56-LIMKi
Lipoxygenase	0.8689	Esculetin
LRRK2	0.1273	PFE-360
LRRK2	0.0449	CZC54252
LRRK2	0.1648	GNE0877

TAK1	1.0262	Takinib
Mitogen-activated protein kinase	0.3384	TLC0816-0600
Mitogen-activated protein kinase	0.5801	5042-0183
Mitogen-activated protein kinase	0.7444	6893-0066
Mitogen-activated protein kinase	0.7831	Y030-3968
Mitogen-activated protein kinase	0.8121	G856-2051
Mitogen-activated protein kinase	0.8315	G420-0124
Mitogen-activated protein kinase	0.8605	2595-1042
Mitogen-activated protein kinase	0.8605	TLC1405-0046
Mitogen-activated protein kinase	0.9088	D220-0956
Mitogen-activated protein kinase	0.9475	6893-0070
Mitogen-activated protein kinase	0.9475	Y070-5049
Mitogen-activated protein kinase	1.0055	D011-6101
Mitogen-activated protein kinase	1.0151	Y030-8119
Mitogen-activated protein kinase	1.0345	Y030-5297
Mitogen-activated protein kinase	1.0487	C498-0579
Mitogen-activated protein kinase	1.0635	D220-1131
Mitogen-activated protein kinase	1.1022	TLC0308-0923
Mitogen-activated protein kinase	1.1215	TLC2557-0175
Mitogen-activated protein kinase	1.1795	E456-0851
Mitogen-activated protein kinase	1.1892	TLC0467-0040
Mitogen-activated protein kinase	1.2762	TLC3407-3786
Mitogen-activated protein kinase	1.2809	C074-0127
Mitogen-activated protein kinase	1.2859	TLC3334-3043
Mitogen-activated protein kinase	1.2955	TLC5123-0125
Mitogen-activated protein kinase	1.3439	TLC2536-1874
Mitogen-activated protein kinase	1.9723	Y020-4911
Mitogen-activated protein kinase kinase kinase 5	1.0635	TLC1588-0718
Mitogen-activated protein kinase kinase kinase 5	1.0925	TLC0271-0012
Mitogen-activated protein kinase kinase kinase 5	0.9185	TLC1414-1209
KSR2	1.0112	APS-2-79
MAP4K4 for Cell; MAP4K4 for Kinase	1.0262	PF6260933
MAP4K4/MINK1/MAP4K6	0.6091	DMX-5084
MEK1; MEK2	0.4061	U0126-EtOH
MEK1; MEK2	0.7566	PD318088
MEK1	0.6891	PD 98059
MEK5	1.2762	GW 284543 hydrochloride
Mitochondrial Metabolism	0.5468	Mitochonic acid 5
MK2	0.5318	MK2-IN-1 hydrochloride
necrosis(MLKL)	3.3333	(E)-Necrosulfonamide
MNK2;MNK1	0.0449	CGP 57380
MNK1	0.3481	TLC2824-0008
mTOR	0.1160	mTOR inhibitor-1
mTOR	0.1573	Temsirolimus
mTORC1; mTORC2	0.3820	KU0063794
TMBIM6	1.1461	TMBIM6 antagonist-1
NF-κB	0.9438	Esculentoside A
NF-κB	1.1236	Wedelolactone
NF-κB	1.1311	Forsythoside B
NOS	0.7865	7-Nitroindazole
SREBP1;SREBP	1.1985	PF 429242
Nrf2	2.3687	TBHQ
cyclin G-associated kinase (GAK)	1.2665	SGC-GAK-1
Death-associated protein kinase 3	1.1022	TLC3385-2049
Death-associated protein kinase 3	1.2085	TLC3223-0019
Death-associated protein kinase 3	1.3149	TLC3223-0018
Histidine kinase	0.6381	TLC0578-0013
IRAK4;cGAS	0.8390	IRAK4-IN-4
Multi-kinase	0.2622	Multi-kinase inhibitor I
Pantothenate kinase 3	1.4405	TLC2726-1127
PCSK9	0.6966	R-IMPP

PCSK9	0.7116	SBC110736
PCSK9	1.2135	SBC115076
penetrant pantothenate kinase (PANK)	0.8390	PZ-2891
Phosphatidylinositol-5-phosphate 4-kinase type-2 alpha	1.1408	TLC0539-1540
Phosphatidylinositol-5-phosphate 4-kinase type-2 alpha	1.2472	TLC2117-0025
Protein kinase Pfmrk	2.2527	TLC1011-0423
sEH	3.0562	AUDA
SPRK1	1.0112	SPHINX
α-KG	1.0925	Disodium (R)-2-Hydroxyglutarate
oxidative phosphorylation (OXPHOS)	0.2022	BAM 15
oxidative phosphorylation (OXPHOS)	0.2697	IM156
P2Y12	1.3034	Piperazine, 1-[(5-methyl-3-isoxazolyl)methyl]- (9CI)
p38 MAPK	0.2846	TA02
p38α	0.2996	R1487
p38α; p38β	0.2996	SB202190
p38	0.2697	PD 169316
PAK1;PAK2;PAK4	0.2472	FRAX1036
PAK1; PAK2 ; PAK3 ;PAK4	0.1573	FRAX486
PAK1; PAK2; PAK2; PAK3	0.2247	FRAX597
PDGFR	0.3371	Sennoside B
PDK1;PDK2;PDK3;PDK4	0.7638	VER-246608
PDK	1.7154	JX06
eIF-2α	0.3190	BTdCPU
eIF-2α phosphatase	0.2547	Sal003
eIF-2α	0.4195	Salubrinal
eIF-2AK1 (HRI); eIF-2AK2 (PKR); eIF-2AK3 (PERK)	1.4382	GSK2606414
PERK	0.3670	Azoramide
Phosphoglycerate kinase	0.8315	TLC0554-0044
Phosphoglycerate kinase	1.3535	TLC0777-0025
Phosphatase	0.9888	NSC 95397
Phosphoglycerate kinase, glycosomal	0.6188	K788-8636
Phosphoglycerate kinase, glycosomal	0.7734	C699-0080
Phosphoglycerate kinase, glycosomal	0.7831	G346-0172
Phosphoglycerate kinase, glycosomal	0.8121	G856-4310
Phosphoglycerate kinase, glycosomal	0.8411	K786-6247
Phosphoglycerate kinase, glycosomal	0.8701	C679-5515
Phosphoglycerate kinase, glycosomal	1.1215	G346-0145
Phosphoglycerate kinase, glycosomal	1.2375	K280-0503
alkaline phosphatase	0.4794	Tracheloside
PI4KIIIβ;PI4KIIIα;PI3Kδ;PI3Kγ;PI3Kα;PI3KC2γ	0.3577	PI4KIIIbeta-IN-9
PI4KIIIβ;PI4KIIIα;PI3Kδ;PI3KC2γ;PI3Kα;PI3Kγ	1.2665	PI4KIIIbeta-IN-10
p110α	0.7348	E781-0728
p110α	0.8895	TLC2031-0726
p110α	0.9088	C712-1180
p110α	0.9861	TLC0225-0023
p110α; p110α; p110β; p110γ; p110δ	0.5918	PIK294
p110α; p110β; p110γ; p110δ	0.1049	PIK293
p110α; p110β; p110γ; p110δ	0.3221	TGX221
p110α; p110γ; p110δ; PI3Kβ	1.4007	A66
p110α; PIKfyve	0.1798	YM201636
p110α;p110β;p110γ;p110δ	0.4641	IPI-3063
p110δ;p110α;p110γ;Vps34;p110β	0.2247	PF4989216
PI3K/mTOR	0.5993	GNE317
PI3KIIIβ	1.0487	BQR695
PI3-kinase p110-delta subunit	0.7831	E947-0749
PI3-kinase p110-delta subunit	1.0538	E947-0640
PI3-kinase p110-gamma subunit	1.4232	8010-3139
PI3Kα	0.5768	GDC0326
PI3Kα	0.8464	HS173

PI3Kα; PI3Kβ; PI3Kγ; PI3Kδ	1.0861	AS604850
PI3Kα; PI3Kβ; PI3Kγ; PI3Kδ	1.0936	AS605240
PI3Kα; PI3Kβ; PI3Kγ; PI3Kδ; Vps34	0.7116	PIK-III
PI3Kβ; PI3Kγ; PI3Kδ	0.8539	CZC24832
PI3Kβ; PI3Kγ; PI3Kδ	1.7079	IC87114
PI3Kγ	0.4419	TASP0415914
PI3Kγ	1.0337	CAY10505
PI3Kγ	1.5955	Myricetin
PI3Kδ	0.7058	Selective PI3Kδ Inhibitor 1
PI5P4Kγ	1.1835	NIH12848
PIKfyve	1.3258	Vacuolin-1
PIKfyve	1.0861	APY0201
Vps34	0.4931	VPS34-IN1
Vps34	1.4682	VPS34 inhibitor 1 (Compound 19, PIK-III analogue)
PI4K	0.4641	KDU691
Serine/threonine-protein kinase PIM1	0.8411	6339-0128
Serine/threonine-protein kinase PIM1	0.8798	TLC3099-6012
Serine/threonine-protein kinase PIM1	1.2085	TLC9995-1727
Pim1	0.6292	SMI-4a
Pim1	0.6667	TCS PIM-1 1
Serine/threonine-protein kinase PIM2	0.8121	TLC0207-0894
Pim1; Pim2	1.0562	SMI-16a
Pim1; Pim2; Pim3	0.4719	CX-6258 HCl
PKA	0.2921	HA-100
PKA	0.5993	8-Bromo-cAMP(sodium salt)
PDK1	1.2809	PS 48
PKC-θ	0.6768	T5817
Protein kinase C zeta	1.1698	TLC0745-0325
Protein kinase C zeta	1.3149	TLC3111-0063
PKC-ι	0.8121	PKC-iota inhibitor 1
PKCα; PKCβ1; PKCβ2; PKCγ; PKCε	0.0075	Ro 31-8220 Mesylate
PKCα; PKCβ; PKCγ; PKCδ; PKCζ	1.4831	Go 6983
PKCα; PKCε	0.7790	Myricetrin
PKCβ	1.0637	PKCβ inhibitor 1
PKC	0.3221	TAS301
PKC	1.1236	Bisindolylmaleimide IV
Pyruvate kinase isozymes M1/M2	1.8756	TLC1741-0144
Pyruvate kinase isozymes M1/M2	2.2172	C325-0361
Pyruvate kinase isozymes M1/M2	2.3880	G017-2156
Serine/threonine-protein kinase PLK1	0.6574	TLC3394-1112
Serine/threonine-protein kinase PLK1	0.7251	TLC0192-0764
Serine/threonine-protein kinase PLK1	0.8121	TLC2590-0776
Serine/threonine-protein kinase PLK1	0.8218	TLC0348-0874
Serine/threonine-protein kinase PLK1	0.8218	TLC0398-0467
Serine/threonine-protein kinase PLK1	0.8218	TLC3139-1169
Serine/threonine-protein kinase PLK1	0.8315	TLC1322-0038
Serine/threonine-protein kinase PLK1	0.8411	TLC0266-1712
Serine/threonine-protein kinase PLK1	0.8701	TLC0698-0238
Serine/threonine-protein kinase PLK1	0.8701	TLC0903-3829
Serine/threonine-protein kinase PLK1	0.8798	TLC0823-0016
Serine/threonine-protein kinase PLK1	0.9185	TLC0030-0020
Serine/threonine-protein kinase PLK1	0.9281	TLC0772-2452
Serine/threonine-protein kinase PLK1	0.9281	TLC0808-0294
Serine/threonine-protein kinase PLK1	0.9378	TLC2590-0102
Serine/threonine-protein kinase PLK1	0.9668	TLC0191-2972
Serine/threonine-protein kinase PLK1	0.9668	TLC3097-2743
Serine/threonine-protein kinase PLK1	0.9861	TLC0526-2250
Serine/threonine-protein kinase PLK1	0.9958	TLC0599-0220

Serine/threonine-protein kinase PLK1	0.9958	TLC1750-0089
Serine/threonine-protein kinase PLK1	1.0151	TLC0698-0181
Serine/threonine-protein kinase PLK1	1.0345	TLC0373-0806
Serine/threonine-protein kinase PLK1	1.0442	TLC0245-0029
Serine/threonine-protein kinase PLK1	1.0442	TLC1958-0047
Serine/threonine-protein kinase PLK1	1.0538	TLC0594-0164
Serine/threonine-protein kinase PLK1	1.0635	TLC1329-0007
Serine/threonine-protein kinase PLK1	1.0635	TLC1589-0100
Serine/threonine-protein kinase PLK1	1.0732	TLC2617-1500
Serine/threonine-protein kinase PLK1	1.1215	TLC1716-0309
PLK1	1.1602	5-ETHYL-4,5,6,7-TETRAHYDRO-THIAZOLO[5,4-C]PYRIDIN-2-YLAMINE
Serine/threonine-protein kinase PLK1	1.1698	TLC0383-0165
Serine/threonine-protein kinase PLK1	1.1698	TLC2712-0142
Serine/threonine-protein kinase PLK1	1.1892	TLC0599-0111
Serine/threonine-protein kinase PLK1	1.1892	TLC1065-0447
Serine/threonine-protein kinase PLK1	1.2085	TLC1018-1554
Serine/threonine-protein kinase PLK1	1.2182	TLC0698-0077
Serine/threonine-protein kinase PLK1	1.2278	TLC1835-0087
Serine/threonine-protein kinase PLK1	1.2375	TLC2704-0045
Serine/threonine-protein kinase PLK1	1.2472	TLC1218-0127
Serine/threonine-protein kinase PLK1	1.2568	TLC1439-0050
PLK1	0.9139	SBE 13 hydrochloride
PLK1; PLK2; PLK3	1.2509	RO 3280
TASK-1	0.4045	ML365
Tyrosine kinase non-receptor protein 2	1.3535	TLC0311-0239
TNK2; TNK2	0.5468	XMD16-5
TNK2; TNK2	1.4232	XMD8-87
PPARα; PPARγ; PPARδ	0.8165	T0070907
PPAR	2.2846	Pseudolaric Acid B
PTEN	0.8024	bpV (HOpic)
PTEN	1.8652	VO-Ohpic trihydrate
B-Raf; B-Raf (V600E); C-Raf-1 (Y340D/Y341D)	0.0524	PLX4720
B-Raf; C-Raf	0.0075	B-Raf IN 1
Serine/threonine-protein kinase B-raf	0.2804	TLC1566-0618
SOS1	0.3867	I-49 free base
SOS1	0.4061	I-37 free base(2359690-13-2(free base))
SOS1	0.4351	BAY-293
Raf	0.7715	Dabrafenib Mesylate
Raf	1.0861	L779450
K-Ras(G12C)	1.0925	ARS-853
K-Ras	0.8015	K-Ras(G12C) Inhibitor 6
K-Ras(G12C)	1.5730	K-Ras(G12C) inhibitor 9
K-Ras(G12C)	1.5952	K-Ras(G12C) inhibitor 12
KRAS G12C	0.6517	6H05 TFA
KRAS G12C	0.9738	1588-A4
KRAS	1.0925	KRAS inhibitor-9
KRAS G12C	1.2060	ARS-1620
KRAS G12C	1.3729	AMG-510
K-Ras	0.7348	K-Ras-IN-1
K-Ras	0.4270	Oncrasin 1
Ras-Raf	0.1573	Kobe2602
Ras-Raf	0.9663	Kobe0065
C-Raf	1.4007	GW5074
C-Raf	0.7341	ZM 336372
Rab7	0.5543	ML098
Rab7	0.9588	CID-1067700

Rho	0.1723	CCG-222740
Rho GEFs	1.6629	Y16
Rac1; Rac1b; Rac2; Rac3	1.1086	EHT 1864
Rac1	1.2509	EHop016
RIPK1	0.6517	Necrostatin 2 racemate
ROCK	0.3970	BDP5290
ROCK	0.5918	Hydroxyfasudil Hydrochloride
ROCK	0.8390	ZINC00881524
ROCK1 (p160ROCK); ROCK2	0.5393	Y-27632 dihydrochloride
ROCK1; ROCK2	0.3296	RKI1313
ROCK1; ROCK2	0.3371	RKI1447
ROCK1; ROCK2	1.3858	GSK429286A
Rho-associated protein kinase 2	0.5414	TLC1105-0149
Rho-associated protein kinase 1	0.9475	TLC2976-0103
ROS	0.1049	Plumbagin
XO	0.7566	4-Methoxy-2-oxo-1,2-dihydro-pyridine-3-carbonitrile
Sphingosine kinase 1	0.0870	TLC1092-1026
Sphingosine kinase 1	0.2030	TLC1092-1030
SphK	0.3745	MHP
SphK	0.5918	CYM5442
SphK1	0.3071	SKI II
S6K1	1.0712	S6K-18
RSK1;RSK2;RSK3	0.1948	LJI308
RSK1; RSK2; RSK3	0.4944	LJH685
p70 S6K	0.1648	PF4708671
SIK1;SIK2;SIK3	0.2996	HG-9-91-01
STK19	0.6442	ZT-12-037-01
Serine Protease	0.7566	Benzamidine HCl
Serine/threonine-protein kinase 33	1.5179	TLC0037-1814
STK33	1.9700	ML281
PKD1; PKD2; PKD3	0.0000	CRT0066101 dihydrochloride
PKD1	0.2172	CID 797718
SRPK1	0.9958	SPHINX31
SRPK1	1.8277	SRPIN340
WNK	1.6704	WNK463
eukaryote protein kinases;tyrosine kinase	1.4082	(+)-Isocorydine hydrochloride
SGK1	0.3771	EMD638683
Serine/threonine-protein kinase Sgk1	1.1215	TLC2173-1125
Src	0.1063	1-Naphthyl PP1 hydrochloride
Lck; Src	0.0300	WH-4-023
Src-PTK	0.6142	KX1004
Tyrosine-protein kinase FYN	1.1795	TLC0873-0006
Fyn; Lyn; Src; Yes	1.2809	SU6656
STAT3	0.2697	STAT3-IN-1
STAT3	0.6292	ML116
STAT5	0.9363	CMD178 TFA
Streptokinase A	0.6091	TLC0646-1826
Streptokinase A	0.6381	TLC0373-1032
Streptokinase A	0.7154	TLC2518-0418
Streptokinase A	0.7348	TLC0642-0069
Streptokinase A	0.7541	TLC1374-0460
Streptokinase A	0.8024	TLC0642-2248
Streptokinase A	0.8411	TLC1305-0368
Streptokinase A	0.9185	TLC0642-3483
Streptokinase A	0.9571	TLC2518-0498
Streptokinase A	0.9668	TLC0642-0058
Streptokinase A	0.9765	TLC0608-0664
Streptokinase A	0.9765	TLC3385-1622
Streptokinase A	0.9861	TLC3226-1387

Streptokinase A	1.0538	TLC1074-0407
Streptokinase A	1.0635	TLC0559-0097
Streptokinase A	1.0732	TLC3385-1489
Streptokinase A	1.1118	TLC2645-0211
Streptokinase A	1.1698	TLC0646-1820
Streptokinase A	1.3149	TLC0642-3344
Streptokinase A	1.3342	TLC1804-0134
Streptokinase A	1.3342	TLC3385-1405
Streptokinase A	0.6188	G370-0148
Streptokinase A	0.7116	8137-0163
Streptokinase A	0.7154	E157-1831
Streptokinase A	0.7348	K844-0806
Streptokinase A	0.7444	3652-0222
Streptokinase A	0.7444	C795-0149
Streptokinase A	0.8798	3966-3242
Streptokinase A	0.8798	C656-0598
Streptokinase A	0.9668	Y020-0731
Streptokinase A	1.0055	C794-0299
Streptokinase A	1.0248	5750-1995
Streptokinase A	1.0538	4576-0111
Streptokinase A	1.1022	5408-2535
Streptokinase A	1.2060	8451-07203
Streptokinase A	1.2182	3948-0328
Streptokinase A	1.2210	C162-0166
Streptokinase A	1.2375	6466-1301
Streptokinase A	1.4792	Y020-0823
Streptokinase A	1.5655	C130-0074
Streptokinase A	1.6404	8009-7155
Syk	0.2097	BAY 61-3606
Tyrosine-protein kinase SYK	1.1698	TLC0849-2247
Syk	1.5955	R406
Syk	0.9213	R406 free base
pan-TAM receptor	0.7640	RU-301
AXL; Mer; Tyro3	0.2547	LDC1267
AXL; Mer; Tyro3	0.2921	UNC2881
TβRI; TβRII	0.3745	LY2109761
TGFβ	0.5094	SJ000291942
TGFβ	0.2772	ITD1
TGF-βRI (ALK5)	0.6966	SD208
bone morphogenetic protein (BMP)	0.4270	SB 4
RIP3	0.3190	HS1371
RIP1; RIP3	0.7865	GSK583
RIP1	0.4569	RIPA-56
RIP1	0.4719	Necrostatin-1
RIP1	0.6671	GSK547
RIP1	0.9571	GSK963 (Racemate)
RIP1	1.2210	GSK481
TOPK	0.3071	OTS964
Trk	0.1049	CH7057288
tyrosine kinase	0.0225	AC1NS4RE
tyrosine kinase	0.0524	Toceranib Phosphate
TrkB	0.2472	7,8-Dihydroxyflavone
TrkB	0.5618	ANA12
TYK2	0.0193	RO495
PTK	0.6742	Tyrphostin AG30(AG30)
PTK	0.5918	ST 271
Protein tyrosine kinase 2 beta	1.1022	TLC3385-1416
Protein tyrosine kinase 2 beta	1.5952	TLC1572-0073
ZAP70	1.5131	ZAP-180013

VEGFR	0.2417	AG-13958
VEGFR	1.3258	Coumarin-3-carboxylic acid
VEGFR2	0.0824	SKLB1002
VEGFR2	0.2397	JK-P3
VEGFR3	0.6442	MAZ51
VEGFR3	0.8240	SAR131675
VEGF-A	1.9101	hVEGF-IN-1
VC	1.7016	Sodium L-ascorbyl-2-phosphate
Wnt	1.1118	IWP2
